# Mechanistic insights into p53‐regulated cytotoxicity of combined entinostat and irinotecan against colorectal cancer cells

**DOI:** 10.1002/1878-0261.13060

**Published:** 2021-07-29

**Authors:** Christian Marx, Jürgen Sonnemann, Mandy Beyer, Oliver D. K. Maddocks, Sergio Lilla, Irene Hauzenberger, Andrea Piée‐Staffa, Kanstantsin Siniuk, Suneetha Nunna, Lisa Marx‐Blümel, Martin Westermann, Tobias Wagner, Felix B. Meyer, René Thierbach, Christina S. Mullins, Said Kdimati, Michael Linnebacher, Francesco Neri, Thorsten Heinzel, Zhao‐Qi Wang, Oliver H. Krämer

**Affiliations:** ^1^ Leibniz Institute on Aging – Fritz Lipmann Institute (FLI) Jena Germany; ^2^ Department of Toxicology University Medical Center Johannes Gutenberg University Mainz Germany; ^3^ Cancer Research UK Beatson Institute Glasgow UK; ^4^ Department of Biochemistry Center for Molecular Biomedicine Institute for Biochemistry and Biophysics Friedrich Schiller University of Jena Germany; ^5^ Department of Paediatric Haematology and Oncology Children's Clinic Jena University Hospital Germany; ^6^ Research Center Lobeda Jena University Hospital Germany; ^7^ Wolfson Wohl Cancer Research Centre Institute of Cancer Sciences University of Glasgow UK; ^8^ Electron Microscopy Center Jena University Hospital Germany; ^9^ Cellular and Molecular Medicine Howard Hughes Medical Institute University of California, San Diego La Jolla CA USA; ^10^ Department of Human Nutrition Institute of Nutrition Friedrich Schiller University of Jena Germany; ^11^ Molecular Oncology and Immunotherapy Department of Thoracic Surgery University of Rostock Germany; ^12^ Molecular Oncology and Immunotherapy Department of General, Visceral, Vascular and Transplantation Surgery University of Rostock Germany; ^13^ Faculty of Biological Sciences Friedrich‐Schiller‐University of Jena Germany

**Keywords:** acetylation, apoptosis, BAK, mitochondria, p53, replication stress

## Abstract

Late‐stage colorectal cancer (CRC) is still a clinically challenging problem. The activity of the tumor suppressor p53 is regulated via post‐translational modifications (PTMs). While the relevance of p53 C‐terminal acetylation for transcriptional regulation is well defined, it is unknown whether this PTM controls mitochondrially mediated apoptosis directly. We used wild‐type p53 or p53‐negative human CRC cells, cells with acetylation‐defective p53, transformation assays, CRC organoids, and xenograft mouse models to assess how p53 acetylation determines cellular stress responses. The topoisomerase‐1 inhibitor irinotecan induces acetylation of several lysine residues within p53. Inhibition of histone deacetylases (HDACs) with the class I HDAC inhibitor entinostat synergistically triggers mitochondrial damage and apoptosis in irinotecan‐treated p53‐positive CRC cells. This specifically relies on the C‐terminal acetylation of p53 by CREB‐binding protein/p300 and the presence of C‐terminally acetylated p53 in complex with the proapoptotic BCL2 antagonist/killer protein. This control of C‐terminal acetylation by HDACs can mechanistically explain why combinations of irinotecan and entinostat represent clinically tractable agents for the therapy of p53‐proficient CRC.

## Graphical abstract

Irinotecan induces post‐translational modification of p53, DNA repair by homologous recombination (HR), and p53 target gene expression. Entinostat and irinotecan combinations increase the C‐terminal acetylation and reduce the N‐terminal phosphorylation of p53, and impair p53 target gene transcription and HR. This drug combination additionally alters the expression and activity of BCL2 proteins and promotes the complex formation of acetylated p53 and BAK. These mechanisms subsequently trigger mitochondrial membrane permeabilization that leads to intrinsic apoptosis.

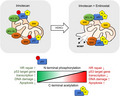

**Abbreviations**
ABT: Navitoclax (ABT‐263)
acK382‐p53: p53 acetylated at its lysine (K) residue 382
BAK: BCL2 antagonist/killer
BAX: BCL2‐associated X protein
BCL: B‐cell lymphoma
CBP: CREB‐binding protein
cl.: cleaved
COAD: colon adenocarcinoma
CPI: CPI‐1612
CRC: colorectal cancer
DBD: DNA‐binding domain
FCS: fetal calf serum
FDA: U.S. food and drug administration
HAT: histone acetyltransferase
HCT116^wt^
: HCT116 cell line expression wild‐type p53 (wt)
HCT116^Δ^ ^p53^
: HCT116 cell line with p53 deletion (Δp53; p53 null; p53‐/‐)
HDAC: histone deactylase
HDACi: HDAC inhibitor
HHC: Hansestadt Hamburg colon cancer cells
HR: homologous recombination
HROC: Hansestadt Rostock colon cancer cells
IgG: immunoglobulin G
IP: immunoprecipitation
Iri: irinotecan
Iri+MS: combined treatment with irinotecan and entinostat
MOMP: mitochondrial outer membrane permeabilization
MS: entinostat (MS‐275)
mt: mitochondrial
p53^6KR^
: acetylation incompetent C‐terminal mutant of p53
p53^K120R^
: acetylation incompetent DBD mutant of p53
PI: propidium iodide
pS15‐p53: p53 phosphorylated at its serine (S) residue 15
PTM: post‐translational modification
READ: rectum adenocarcinoma
RS: replication stress
TCGA: The Cancer Genome Atlas
TEM: transmission electron microscopy
TOP1: toposiomerase‐1
γH2AX: phosphorylated histone protein H2AX
Δp53‐EV: p53 null HCT116 cell line reconstituted with an empty vector (EV) construct
Δp53‐p53^6KR^
: p53 null HCT116 cell line reconstituted with p53^6KR^
ΔΨ_M_
: mitochondrial membrane potential

## 1 Introduction

Post‐translational modifications (PTMs) control major protein functions and activities. Lysine acetylation of proteins was discovered more than 50 years ago, and recent evidence shows that it is one of the most important PTMs *in vivo* [1]. Histone deacetylases (HDACs) remove acetyl groups from proteins and are divided into four phylogenetic classes in mammals. Eleven zinc‐dependent enzymes form classes I, II, and IV, and seven NAD‐dependent sirtuins are class III [1]. It has become clear that HDAC inhibitors (HDACi) will only develop their therapeutic potential in combination treatments [2, 3].

Colorectal cancer (CRC) is the second most common cause of cancer death. Alone in the United States, approximately 147 950 individuals will be diagnosed with CRC and 53 200 will die from the disease in 2020 [4]. Thus, improved therapies should be identified for this unmet clinical need. Due to aberrant HDAC activities in such tumors, HDACi are tested against cancer, including CRC. All clinically relevant inhibitors block class I HDACs [5, 6, 7]. From a therapeutic perspective, it is promising that HDACi sensitize tumor cells to chemotherapeutics causing replication stress (RS) and DNA damage [8, 9, 10]. There is an ongoing intense search for reliable markers and molecular mechanisms that indicate whether a cell is responsive to such treatment.

The tumor suppressor p53 modulates the expression of genes for cell cycle arrest, DNA repair, and apoptosis. This allows p53 to control cell fate upon disturbed DNA replication and damage. Cell cycle arrest and DNA repair prevail until irreparable or continuous damage drives the accumulation of proapoptotic p53 target genes [11, 12, 13]. Two main pathways initiate apoptosis. The intrinsic pathway is activated by mitochondrial membrane permeabilization (MOMP), and the extrinsic pathway is triggered by receptor signaling [14]. Multiple PTMs, including phosphorylation by checkpoint kinases and acetylation by histone acetyltransferases (HATs), regulate p53 and increase the transcription of proapoptotic proteins [13, 14, 15, 16, 17]. The relative contributions of an imbalanced pro‐ and antiapoptotic BCL2 protein expression and of p53 hyperacetylation by HDACi toward tumor cell apoptosis are not fully deciphered [13, 17, 18]. Regarding the interactions of p53 with BCL2 proteins, it is known that the acetylation of the p53 DNA‐binding domain (DBD), which lies in the middle of the protein, promotes interactions with the proapoptotic pore‐forming proteins BCL2‐associated X protein (BAX) and BCL2 antagonist/killer (BAK) [15, 16, 17, 19, 20]. However, despite the well‐known relevance of p53 C‐terminal acetylation for gene regulation, it is unclear whether this acetylation of p53 can regulate MOMP.

Here, we provide new insights into how p53, class I HDACs, and CREB‐binding protein (CBP)/p300 act as key gatekeepers for the survival of cells with RS. We used wild‐type p53‐positive (HCT116^wt^) and p53‐negative (HCT116^Δp53^) human CRC cell lines, human primary short‐term CRC cell cultures, and patient‐derived CRC organoids to measure cellular responses to the topoisomerase I (TOP1) inhibitor irinotecan in combination with the class I HDACi entinostat (MS‐275) and the CBP/p300 inhibitor CPI‐1612. Irinotecan is an FDA‐approved drug to treat CRC. It causes torsional DNA damage and RS in S phase and stalls cells in G2 phase. DNA strand breaks upon prolonged TOP1 inhibition are repaired by homologous recombination (HR). Unfortunately, irinotecan frequently produces severe side effects that lead to a discontinuation of its use in patients with cancer. Combined treatment approaches could allow a reduction in the irinotecan doses that are applied to patients [21, 22, 23]. The well‐tolerated epigenetic drug entinostat is tested in phase III clinical trials for the treatment of various tumor entities [6, 18]. CPI‐1612 is a new inhibitor of CBP and p300 [24]. We show that the combined application of irinotecan and entinostat synergistically kills CRC cells *in vitro* and *in vivo* and that the induction of C‐terminal hyperacetylation of p53 is an absolute requirement for apoptosis induction by irinotecan and entinostat.

## 2 Methods

### 2.1 Cell culture

HCT116 cells were a gift from B. Vogelstein (Baltimore, USA). H1299 cells with inducible p53^wt^ or p53^K120R^ were kindly provided by S. B. McMahon (Philadelphia, USA) [25]. RKO ATCC cells were from the DSMZ Braunschweig and a gift from M. Zörnig (Frankfurt/Main, Germany). DNA fingerprinting analysis using eight different, highly polymorphic short tandem repeat loci confirmed cell authenticity (conducted by the Leibniz Institute DSMZ, Braunschweig, Germany). Samples were tested negative for mitochondrial DNA from rodent cells (mouse, rat, hamster). Cells were maintained in high glucose (4.5 g·L^−1^) DMEM with stable glutamine, 10% fetal calf serum (FCS), and 250 µg·mL^−1^ gentamycin (Thermo Fisher Scientific, Waltham, MA, USA). HROC/HHC (Hansestadt Rostock/Hansestadt Hamburg colon cancer) cells were isolated from excised CRCs according to Ref. [26]. Low‐passaged cell lines were maintained in DMEM containing 10% FCS (Pan Biotech, Aidenbach, Germany). A. Poth (Roßdorf, Germany) kindly provided BALB/c‐3T3‐A31‐1‐1 cells from Hatano Research Institute of Japan. These were maintained in DMEM/HAM's F‐12 (3.0 g·L^−1^ glucose; Biochrom, Berlin, Germany), 5% FCS, and 100 U·mL^−1^ penicillin/streptomycin. Cells between passages 20–40 were used for the BALB transformation assay (˜ 70% confluence). All cells were cultivated in a humidified incubator at 37 °C with 5% CO_2_.

### 2.2 Reagents

Irinotecan hydrochloride (CPT‐11) and propidium iodide (PI) were purchased from Sigma‐Aldrich (Deisenhofen, Germany). Entinostat (MS‐275), navitoclax (ABT‐263), and z‐VAD‐FMK (zVAD) were purchased from Selleck Chemicals (Houston, TX, USA). 3,3′‐dihexyloxacarbocyanine iodide (DiOC_6_(3)) was purchased from Thermo Fisher Scientific. CPI‐1612 [24] was kindly provided from M. Trojer (Constellation Pharmaceuticals, Cambridge, MA, USA). DMSO was used as treatment control.

All other chemicals were bought from Carl Roth GmbH & Co KG (Karlsruhe, Germany). All buffers and solutions were prepared with double‐distilled water (ddH_2_O).

### 2.3 Treatment of cells

Cells were treated with 1–10 µm irinotecan, 1–2 µm entinostat, 20 µm zVAD, 500 nm ABT‐263, 25–1000 nm CPI‐1612, and their combinations for up to 48 h. If indicated, cells were irradiated with 2 Gy using ^137^Cs γ‐irradiation source (Gammacell 40, Nordion, Ottawa, ON, Canada).

### 2.4 Transfection of cells

Cells were transfected with 1 µg of empty vector (EV, pcDNA3.1), p53^wt^ (pRK5‐Flag), and p53^6KR^ (pRK5‐Flag) plasmids using 3 µL Lipofectamine 2000 (in Opti‐MEM; Thermo Scientific) per well. p53^wt^ and p53^6KR^ plasmids were kindly provided by W.‐G. Zhu (Shenzen, China). To obtain p53‐negative cells with a reconstitution of p53 isoforms, the cells were transfected using 3 µL Lipofectamine 2000, 1 µg DNA and 0.1 µg of a nontargeted shRNA (pSUPER) (in Opti‐MEM; Thermo Scientific) per well. The empty shRNA vector contains a puromycin‐resistance cassette that allowed the selection of transfected clones. On the next day, the medium was changed and 0.1 µg·mL^−1^ puromycin (Invivogen, San Diego, CA, USA) was added. Every 2–3 days, the puromycin‐containing medium was changed. After 2 weeks of selection, cells were separated for the first time and the puromycin concentration was increased to 1 µg·mL^−1^. After another 2 weeks, cells were grown in T25 cell culture flasks and DMEM containing 1 µg·mL^−1^ puromycin. After 6 weeks of selection, cells stably kept p53 expression in DMEM without puromycin. Transfection efficiency and persistence of the reconstitution with p53 were controlled via immunoblot.

### 2.5 Human organoid culture

Human intestinal crypts from either tumor tissue or adjacent healthy tissue were isolated and cultured as described in Ref. [27] and seeded (100 organoids/well) in Labtec (Eschelbronn, Germany) chamber slides containing 20 μL of matrigel matrix and 300 μL of growth medium. Both healthy and tumor organoids were sequenced, the tumor organoids harbor mutations in the following genes: *ARID1A, PIK3CA, APC, KRAS,* and *AMER1,* but are wild‐type for *TP53*. The human organoid study has been approved by the Jena University Hospital Ethics Committee (#5032‐01/17).

After 24 h of initial growth, organoids were treated with irinotecan–entinostat combinations for an additional 24 h. Organoid images were captured using a Zeiss AxioVert 40 CFL microscope and an AxioCam MRc 5 (Carl Zeiss, Oberkochen, Germany); live and dead organoids were counted according to their morphological appearance.

### 2.6 *In* *vivo* experiments

Animals used in these experiments were 6‐ to 8‐week‐old male or female NMRI‐Foxn1nu mice (16–25 g body weight). The breeding was done in the animal facility of the Rostock University Medical Center under specified pathogen‐free conditions. Standard pellet food and water *ad libitum* were given during exposition to 12‐h light/12‐h darkness cycles. The tumor specimens (3 × 3 × 3 mm) were incubated in matrigel (Corning, Kaiserslautern, Germany) for > 10min at 4 °C prior to flank implantation under anesthesia (ketamine/xylazine, 90/6 mg·kg^−1^ body weight). Therapy was initiated upon tumor establishment (4–5 mm diameter). All mouse experiments were performed according to the guidelines of the local animal use and care committee (Landesamt für Landwirtschaft, Lebensmittelsicherheit und Fischerei Mecklenburg‐Vorpommern, permit number: LALLF M‐V/TSD/7221.3‐1‐063/18).

The mice were randomized into two different therapy groups: (a) control [100 µL 5% DMSO (AppliChem GmbH, Darmstadt, Germany) in 0.9% NaCl (B. Braun Melsungen AG, Melsungen, Germany), intraperitoneal (i.p.), thrice weekly, six times in total]; and (b) combination of entinostat (2.5 mg·kg^−1^ bw in 5% DMSO in 0.9% NaCl, orally, five times weekly, 15 times in total) and irinotecan (20 mg·kg^−1^ bw in 5% DMSO in 0.9% NaCl in 100 µL total volume, i.p., thrice weekly, nine times in total). Tumor growth and animal behavior were monitored daily. Animals were sacrificed after therapy completion, and tumors and livers were resected, weighed, and photographed.

### 2.7 Flow cytometric analysis of apoptosis, cell death, and mitochondrial transmembrane potential (ΔΨ_M_)

These analyses were performed as previously described in Ref. [28].

### 2.8 β‐Galactosidase staining

Cellular senescence was measured using the senescence β‐galactosidase staining kit (Cell Signaling Technologies, Danvers, MS, USA) following the manufacturer's instruction. Images were examined using a Zeiss AxioVert 40 CFL microscope and an AxioCam MRc 5 (Carl Zeiss).

### 2.9 Whole cell lysates and coimmunoprecipitations

Cell lysates were prepared as described in Ref. [29]. Protein quantification was done by BCA assay (Thermo Scientific). Coimmunoprecipitation was done as described in Ref. [30] using 1 µg of anti‐BAK or anti‐BCL‐XL (Cell Signaling) primary antibodies and rabbit‐IgG (Abcam, Cambridge, UK) as negative controls. Veriblot HRP‐coupled secondary antibodies (Abcam) were used to reveal the proteins.

### 2.10 Mitochondrial isolation and fractionation of mitochondria using carbonate treatment

After harvesting by scraping, cells were resuspended in homogenization buffer [10 mm Tris/HCl (pH 6.7), 10 mm KCl, 150 mm MgCl_2_] and incubated 10 min on ice. Cells were homogenized on ice, and sucrose was added to the suspension (final concentration 0.25 m). Cellular membrane fragments and nuclei were removed by two centrifugation steps at 1000 ***g***. Mitochondria were pelleted at 13 000 ***g*** for 10 min afterward. The supernatant (cytosolic fraction) was removed, and mitochondria were resuspended in homogenization buffer and briefly sonicated or used for further fractionation.

Mitochondrial subfractionation was performed as described in Ref. [31]. Isolated mitochondria were diluted 50‐fold with 100 mm sodium carbonate pH 11.5 and incubated on ice for 30 min. The suspension was centrifuged at 4 °C for 15 min at 20 000 ***g***. After removing the supernatant, containing soluble mitochondrial matrix proteins, the membrane pellet was resolved in homogenization buffer (see above). The pH of the solutions was adjusted to 7 using 1 m HCl afterward.

### 2.11 Immunoblotting

Sample preparation was done as described in Ref. [29]. Antibodies used (Table 1):

**Table 1:** List of primary antibodies used in this study.

Antigen	Dilution	Company
p53 (DO‐1)	1 : 5000	Santa Cruz Biotechnology Inc., Heidelberg, Germany
MDM2	1 : 1000	
BAX	1 : 1000	
RAD51	1 : 1000	
p16	1 : 2500	
p19	1 : 1000	
BRCA1	1 : 100	
Cyclin B1	1 : 1000	
Cytochrome *c*	1 : 2500	
p21	1 : 2500	Abcam, Cambridge, UK
Acetyl‐lysine 381 p53	1 : 2500	
Acetyl‐lysine 373 p53	1 : 1000	
Acetyl‐lysine 305 p53	1 : 1000	
RPA32	1 : 2500	
Phospho‐serine 10 histone H3	1 : 2500	Merck Millipore, Darmstadt, Germany
Acetyl‐histone H3	1 : 5000	
ATM	1 : 1000	Novus Biologicals, Centennial, CO, USA
Phospho‐serine 33 RPA32	1 : 2500	Bethyl Laboratories, Montgomery, TX, USA
PARP1	1 : 1000	Cell Signaling, Frankfurt/Main, Germany
BIM	1 : 2500	
BAK	1 : 2500	
BCL‐XL	1 : 5000	
ATR	1 : 1000	
Phospho‐serine 428 ATR	1 : 2500	
Phospho‐serine 1981 ATM	1 : 1000	
Cleaved caspase‐3	1 : 1000	
Phospho‐serine 15 p53	1 : 2500	
Acetyl‐lysine 382 p53	1 : 1000	

Equal loading of protein was verified by the detection of α‐tubulin (1 : 10 000; Sigma‐Aldrich), HSP90 (1 : 10 000), TOM40 (1 : 10 000), β‐actin (1 : 2000), vinculin (1 : 5000) (from Santa Cruz Biotechnology Inc.), or ATP5A (1 : 5000; Abcam). Densitometric quantification was done with imagej (National Institutes of Health, Bethesda, MD, USA).

### 2.12 Quantitative real‐time PCR

Total RNA was isolated using the Peqgold Total RNA Kit including DNase digestion (Peqlab, Erlangen, Germany). RNA was transcribed into cDNA using Omniscript (Qiagen, Hilden, Germany). Quantitative PCR for *BBC3*, *CDKN1A*, *HDM2*, *BAX, BIM, RAD51, TP53I3, BRCA1, BRCA2, FANCD2, BCL2, BCL2L1, BAK1, BID, 18S,* and *B2M* was performed using the Applied Biosystems 7900HT Real‐Time PCR system (Darmstadt, Germany). Expression levels were normalized to β2‐microglobulin or 18S rRNA. Reactions were done in duplicate using Applied Biosystems Gene Expression Assays [*BBC3* (PUMA): Hs00248075_m1, *CDKN1A* (p21): Hs00355782_m1, *HDM2*: Hs99999008_m1, *BAX*: Hs00180269_m1, *TP53I3* (PIG‐3): HS00936520_m1, *BCL2L11* (BIM): Hs00708019_s1, *RAD51*: Hs00947967_m1, *BRCA1*: Hs01556193_m1, *BRCA2*: Hs00609073_m1, *FANCD2*: Hs00276992_m1, *BCL2*: Hs00608023_m1, *BCL2L1* (BCL‐XL): Hs00236329_m1, *BAK1*: Hs 00832876_m1, *BID*: Hs00609632_m1, *18S* (18S rRNA): Hs99999901_s1, *B2M* (β‐2‐microglobulin): Hs00187842_m1], and Universal PCR Master Mix. All procedures were performed according to the manufacturers' protocols. The relative gene expressions were calculated by the $2^{\left(‐\Delta \Delta C_{t}\right)}$ method.

### 2.13 BALB/c cell transformation assay

This assay was performed as described in Ref. [32].

### 2.14 Fluorescence microscopy

Cells were fixed with 4% paraformaldehyde (in PBS; Carl Roth) and incubated in blocking solution (BS: 1% BSA, 0.4% Triton X‐100 in PBS). Following, samples were incubated in primary antibody solutions against H2AX phosphorylated at serine‐139 (γH2AX; 1 : 1000 in BS; Merck Millipore), 53BP1 (1 : 500 in BS; Novus Biologicals), or RAD51 (1 : 250 in BS; Santa Cruz), washed in PBS and incubated in AlexaFluor 555‐conjugated anti‐rabbit (1 : 500 in BS; Abcam) or AlexaFluor 647‐conjugated anti‐mouse (1 : 500 in BS; Thermo Fisher) secondary antibody solutions. Afterward, samples were stained with 1 µg·mL^−1^ DAPI (Sigma‐Aldrich) and mounted with ProLong Gold Antifade Mountant (Thermo Scientific). Images were examined using a Zeiss AxioImager ApoTome microscope (structured illumination) (Carl Zeiss). zen 2.0 lite software (Carl Zeiss) was used for image analysis.

### 2.15 Transmission electron microscopy

Cells were fixed with 2.5% glutaraldehyde in cacodylate buffer (0.1 m, pH 7.4), washed with cacodylate buffer, carefully harvested by scraping, and fixed within osmium tetroxide (1% in cacodylate buffer). During ascending ethanol series, samples were stained with 2% uranyl acetate. The samples were embedded in Araldite resin (Plano, Wetzlar, Germany) according to the manufacturer's instruction. Ultrathin sections of 50 nm thickness were cut using an Ultracut E ultramicrotome (Reichert–Jung, Wien, Austria) and mounted on Formvar/Carbon coated grids (100 Mesh, Quantifoil Micro Tools GmbH, Jena, Germany). Finally, the ultrathin sections were stained with lead citrate and photographically imaged in a Zeiss EM900 transmission electron microscope (Carl Zeiss).

### 2.16 Freeze‐fracture replica immunogold labeling electron microscopy of p53

Sample preparation was done as described in Ref. [33]. The freeze‐fracture replicas were imaged in a Zeiss EM902A transmission electron microscope (Carl Zeiss). Images were recorded with a 1k (1024 × 1024) FastScan‐CCD‐camera (CCD‐camera and the acquisition software emmanu4 v 4.00.9.17, TVIPS, Munich, Germany).

### 2.17 PTM analysis by mass spectrometry

#### 2.17.1 Immunoprecipitation of p53

Protein G Dynabeads (Thermo Scientific) were cross‐linking with an equal volume of anti‐p53 primary antibodies [50 : 50 (v/v) DO‐1 and Bp53‐12; Santa Cruz] with BS^3^ (Thermo Scientific) following the manufacturers' instructions. Cells were harvested by scraping in lysis buffer (50 mm Tris pH 7.5, 1 mm EDTA, 1% (v/v) NP‐40, 50 mm NaF, 5 mm sodium pyrophosphate, 0.27 m sucrose supplemented with protease inhibitor complete (Roche, Basel, Switzerland), 10 µm trichostatin A, 10 mm sodium butyrate, 10 mm nicotinamide, 1 mm sodium orthovanadate, 10 mm sodium β‐glycerophosphate) followed by brief sonication. Each lysate was incubated with 30 µL of bead‐antibody mixture overnight at 4 °C. Afterward, beads were washed twice with lysis buffer, once with high salt buffer (20 mm Tris pH 8.1, 500 mm NaCl, 0.1% SDS, 1% Triton X‐100, 1 mm EDTA), and thrice with TBST (0.05% Tween‐20 in TBS). Precipitated p53 was eluted from the beads for 10 min at 95 °C in 50 μL 1× LDS buffer (Thermo Scientific). Supernatants were transferred into new tubes, and DTT was added (final concentration 20 mm). Samples were boiled 10 min at 95 °C, separated by SDS/PAGE, and stained with Coomassie.

#### 2.17.2 In‐gel proteolytic protein digestion

The region containing p53 was excised from an SDS gel and washed twice with 50 mm NH_4_HCO_3_, and once 50 mm NH_4_HCO_3_ with 50% acetonitrile. Proteins within the gel slices were reduced using 10 mm DTT (54 °C, 30 min) and subsequently alkylated with 55 mm iodoacetamide (RT, 45 min). Gel pieces were washed with 50 mm NH_4_HCO_3_, and 50 mm NH_4_HCO_3_ with 50% acetonitrile and finally dehydrated using acetonitrile before drying in a SpeedVac. Trypsin (5 μg·mL^−1^ trypsin gold; Promega GmbH (Walldorf, Germany); in 25 mm ammonium bicarbonate) was added and incubated for 12 h at 30 °C. Tryptic peptides were extracted from gel pieces with two 50% (v/v) acetonitrile/water washes and subsequently dried in a SpeedVac.

#### 2.17.3 Liquid chromatography

The tryptic digest obtained was separated by nanoscale C18 reverse‐phase liquid chromatography performed on an EASY‐nLC II (Thermo Scientific) coupled to a Linear Trap Quadrupole—Orbitrap Velos mass spectrometer (Thermo Fisher). Elution was carried out using a binary gradient with buffer A: 2% acetonitrile and B: 80% acetonitrile, both containing 0.1% of formic acid. Samples were resuspended and loaded with buffer A, on a precolumn NS‐MP‐10 BioSphere C18 5 μm 120 Å– 360/100 μm × 0.2cm of length (NanoSeparations), and washed with 25 µL of buffer A at a maximum pressure of 200 bar. Desalted peptides were subsequently eluted at 200 nL·min^−1^ flow, into a 20 cm fused silica emitter (New Objective) packed in‐house with ReproSil Gold 200 C18, 3 μm resin (Dr. Maisch GmbH, Ammerbuch, Germany). The gradient used started at 5% of buffer B and was increased to 28% over 42 min and then to 45% over 13 min. Finally, a washing step at 100% of B was carried out over 10 min followed by a 5‐min re‐equilibration at 5% B for a total duration of 70 min. The eluting peptide solutions were automatically (online) electrosprayed into the mass spectrometer via a nanoelectrospray ion source. An Active Background Ion Reduction Device (ABIRD) was used to decrease the contaminant signal level.

#### 2.17.4 Data‐dependent acquisition

General mass spectrometric conditions were as follows: Spray voltage, 2.4 kV, and ion transfer tube temperature, 200 °C. The mass spectrometer was operated in positive ion mode and used in data‐dependent acquisition mode (DDA). A full scan (FT‐MS) was acquired at a target value of 1 000 000 ions with resolution *R* = 60 000 over a mass range of 350–1600 amu. The top 10 most intense ions were selected for fragmentation in the linear ion trap using Collision Induced Dissociation (CID) using a maximum injection time of 25 ms or a target value of 5000 ions. Multiply charged ions from two to five charges having an intensity greater than 5000 counts were selected through a 1 amu window and fragmented using a normalized collision energy of 35 for 10 ms. Former target ions selected for MS/MS were dynamically excluded for 30 s.

#### 2.17.5 Parallel reaction monitoring

Parallel reaction monitoring acquisition (PRM) was used for the analysis of p53 acetylated peptides. A full scan (FT‐MS) was acquired at a target value of 1 000 000 ions with resolution *R* = 60 000 over a mass range of 350–1600 amu followed by nine PRM scans targeting the molecular masses of acetylated p53 peptides present in the library and peptide 343–351 used for normalization (Table 2). The precursor masses in the PRM scans were fragmented using collision‐induced dissociation in the linear ion trap. Isolation width of 1 *m/z*, the activation time of 10 ms, and normalized collision energy of 36 were used to fragment a maximum target of 5000 ions (or injection time of 25 ms). PRM scans mass ranges were adjusted according to the masses of the monitored parent peptides. PRM data were collected in triplicate.

**Table 2:** Detected p53 fragments after tryptic digestion with corresponding acetylation site, charge, and *m/z*.

p53 peptide	Sequence	Ac. position	*m/z*	*z*	Top 5 Monitored MS/MS peptide fragment	DotP
292–305	(K)**K**GEPHHELPPGSTK(R)	292	519.2670	3	y6, y9^++^, y11^++^, y13^++^, b8^++^	0.96
292–306	(K)KGEPHHELPPGST**K**R(A)	305	571.3007	3	y6, y7^++^, y9^++^, b8, b9^++^	0.86
307–320	(R)ALPNNTSSSPQP**K**K(K)	319	755.8966	2	y3, y5, y7, y12^++^, b11	0.93
307–320	(R)ALPNNTSS**S**PQP**K**K(K)	319	795.8798	2	y3, y5, y11^++^, y12^++^, b11	0.93
320–333	(K)K**K**PLDGEYFTLQIR(G)	321	875.4803	2	y9, y10, y12, y13^++^, b5 (Rt = 51.7)	0.92
320–333	(K)**K**KPLDGEYFTLQIR(G)	320	875.4803	2	y9, y10, y12, y13^++^, b5 (Rt = 50.8)	0.97
343–351	(R)ELNEALELK(D)	none	529.7900	2	y3, y4, y5, y6, y7	0.98
380–386	(R)H**KK**L**M**FK(T)	381–382	516.2890	2	y5, y6, y6^++^, b2, b5	1.00
382–386	(K)**K**L**M**FK(T)	382	362.7067	2	y2, y3, y4, b2, b3	0.97

#### 2.17.6 Data‐dependent analysis

Raw data were processed with Raw2MSN [34], and Mascot generic files generated were analyzed using mascot (Matrix Science Ltd, London, UK, version 2.4.1), querying both: SwissProt database (release 2014_01, restricted to *Homo sapiens*, 20 273 entries) and an in‐house database containing common proteomic contaminants and main isoforms of human p53. Mascot was searched assuming the digestion enzyme trypsin allowing for two miscleavages with a fragment ion mass tolerance of 0.5 Da and a parent ion tolerance of 10 p.p.m. Cysteine carbamidomethylation was specified in Mascot as a fixed modification. Acetylation of protein N termini and lysine residues, oxidation of methionine moieties, and phosphorylation of serine and threonine residues were specified in Mascot as variable modifications. scaffold (version 4.3.2; Proteome Software Inc., Portland, OR, USA) was used to validate MS/MS‐based peptide and protein identifications. Peptide identifications were accepted if they could be established at greater than 95.0% probability as specified by the Peptide Prophet algorithm, resulting in a peptide false discovery rate of 0.02% [35]. Only acetylated peptides still present after filtering were used to build the library used in PRM experiments.

#### 2.17.7 Parallel reaction monitoring and quantitation analysis

Normal and acetylated p53 tryptic peptides precursor and fragment masses, used to setup PRM acquisition methods, were deduced from a library generated using data‐dependent analysis data gathered from p53 immunoprecipitations. The PRM data traces of p53 acetylated tryptic peptides were used for MS/MS‐based quantitation. Raw data were imported into Skyline, and y” and b product ions chromatograms of 1+ and 2+ charges were extracted, summed, and integrated. Only fragment ion peaks with the same retention time were used for quantitation. MS/MS filtering in Skyline was applied to all replicates using: ‘Targeted’ acquisition method, product mass analyzer ‘QIT’ and resolution of 0.6 *m/z*. All matching scans were included in data extraction. Ion series extracted were compared using a 0.5 *m/z* tolerance to the 10 most intense product ions of p53 acetylated peptides library; and dotp values [36] obtained are reported in Table 2.

### 2.18 Database analysis

Gene expression data and correlation coefficients between gene expression and drug sensitivity in NCI‐60 cell line panel were exported using CellMiner web tool [37]. Gene expression of TCGA dataset was analyzed using Gene_DE module in Timer web platform [38].

### 2.19 Statistical analysis

Statistical analyses were done with Microsoft Excel using a two‐tailed Student's *t*‐test or with graphpad (GraphPad Software, San Diego, CA, USA) using one‐way ANOVA test (*P < 0.05, **P < 0.01, ***P < 0.001). Error bars show standard error of the mean (SEM) if not mentioned otherwise.

## 3 Results

### 3.1 Combinations of irinotecan and entinostat induce apoptosis of p53‐proficient cells

**Fig. 1:**
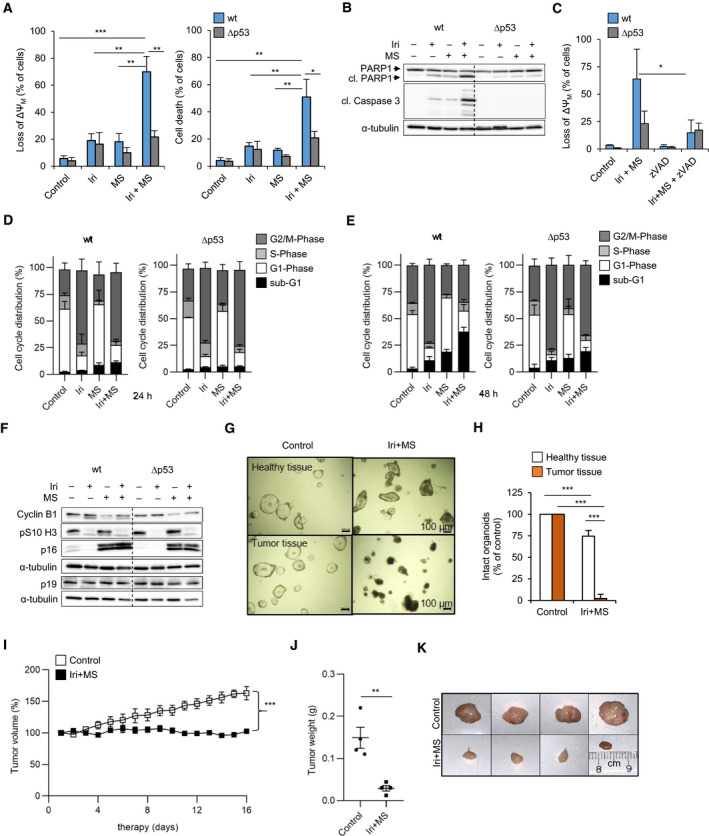
Irinotecan plus entinostat induces apoptosis in p53‐proficient CRC cells, CRC organoids, and established CRC tumors. HCT116^wt^ and isogenic HCT116^Δp53^ cells were exposed to 10 µm irinotecan (Iri), 2 µm entinostat (MS, short for MS‐275, the alterative name of entinostat), and their combination (Iri+MS). (A) The percentages of cells with disrupted mitochondrial transmembrane potential (loss of ΔΨ_M_) and dead cells (accumulation of PI) were quantified by flow cytometry after 48 h. (B) Immunoblot shows the levels of the indicated proteins after 48 h; α‐tubulin verifies equal protein loading (cl., cleaved). (C) Cells were 1 h pretreated with 20 µm zVAD alone ± Iri+MS for 48 h. Loss of ΔΨ_M_ was quantified by flow cytometry. (D, E) HCT116^wt^ and HCT116^Δp53^ cells were exposed to 5 µm Iri ± 2 µm MS. The cells were tested for nuclear PI contents that mirrors cell cycle distributions after 24 h (D) and 48 h (E) by flow cytometry. (F) Expression levels of indicated proteins were analyzed by immunoblot after 24‐h treatment with 10 µm Iri ± 2 µm MS. α‐tubulin serves as a loading control. Due to the similar sizes of p16 and p19, these were probed on individual membranes. (G) Human organoid cultures from healthy and tumor tissue of one patient with known p53 status were exposed for 24 h to 10 µm Iri + 2 µm MS. The amount of intact, living organoids was assessed by microscopy (H). All graphs show the mean value of three independent experiments ± SEM; immunoblots are representative for three independent experiments. (I) 6‐ to 8‐week‐old NMRI‐Foxn1nu mice were treated with solvent (control) or 20 mg·kg^−1^ irinotecan in combination with 2.5 mg·kg^−1^ MS‐275 for 16 days. Changes in tumor volume were monitored daily. The graphs show the mean value of 4 control and 6 Iri+MS‐treated tumors ± SEM; statistic analysis here was done with one‐way ANOVA. (J) The weight of resected tumors was documented after 16 days, and representative pictures are shown in (K). Significances for this figure are: *P < 0.05; **P < 0.01; ***P < 0.001 using two‐tailed *t*‐tests in (A), (C), (H), and (J) or one‐way ANOVA test in (I).

We studied how irinotecan, entinostat, and their combination affect CRC cells. We measured the loss of the mitochondrial membrane potential (ΔΨ_M_), indicating MOMP, and propidium iodide (PI) uptake, a marker for cell death, by flow cytometry [29]. We incubated HCT116^wt^/HCT116^Δp53^ cells with 1–10 µm irinotecan and 1–2 µm entinostat. These doses cover their therapeutic ranges in patients [21, 23]. Several combinations produced synergistic cytotoxic effects (CI < 1; Fig. S1A,B, Table S1). Based on these data, we chose 2 µm entinostat and 5–10 µm irinotecan for our following experiments. Ten micromolar irinotecan or 2 µm entinostat caused up to 20% of ΔΨ_M_ loss and 20% PI‐positive cells in HCT116^wt^ and HCT116^Δp53^ cells. Irinotecan plus entinostat significantly increased the ΔΨ_M_ loss to 70% (*P* < 0.001) and cell death to 60% (*P* < 0.01) in HCT116^wt^ cells (Fig. 1A) but was hardly more effective than the single agents in HCT116^Δp53^ cells (Fig. 1A, Fig. S1A,B). An analysis of short‐term cultured patient‐derived CRC cells with wild‐type p53 (HROC24, HROC113, HHC6548) confirmed that a combined treatment with irinotecan plus entinostat was superior over the single drugs (Fig. S1C).

Immunoblot analyses showed that irinotecan plus entinostat induced the accumulation of cleaved, active caspase 3, and the cleavage of its target PARP1 [29] in HCT116^wt^ cells (Fig. 1B). The pan‐caspase inhibitor zVAD decreased the ΔΨ_M_ loss due to such treatment by 49% in HCT116^wt^ cells (*P* < 0.05) (Fig. 1C, Fig. S1D), confirming caspase‐dependent apoptosis.

Flow cytometry analyses for cell cycle alterations showed that after 24 h, irinotecan alone and combined with entinostat induced an accumulation of cells in the G2/M phase in HCT116^wt^ and HCT116^Δp53^ cells (Fig. 1D). Entinostat p53 independently increased G1 and decreased S phase cell populations and increased sub‐G1 fractions (cells with DNA content < 2N). Sub‐G1 fractions increased to 11% in HCT116^wt^ cells (*P* < 0.001) and remained unaltered in HCT116^Δp53^ cells (*P* < 0.01 to HCT116^wt^) that were exposed to both agents. After 48 h, entinostat further reduced S phase populations in HCT116^wt^ (3%; *P* < 0.001) and HCT116^Δp53^ cells (6%; *P* < 0.01) (Fig. 1E). Irinotecan increased G2/M (HCT116^wt^ 73%; HCT116^Δp53^ 79%; both *P* < 0.001) and decreased G1 phase populations (HCT116^wt^ 11.6%; *P* < 0.001; *P* < 0.01 to HCT116^Δp53^ 6%). Irinotecan plus entinostat caused an accumulation of 38% of HCT116^wt^ cells in sub‐G1 (*P* < 0.001; *P* < 0.01 to HCT116^Δp53^) and fewer cells arrested in G2/M (35%; *P* < 0.001 to HCT116^Δp53^) than upon irinotecan treatment. Irinotecan alone and combined with entinostat caused similar effects in HCT116^Δp53^ cells.

Consistent with these flow cytometry data, we noted that irinotecan increased and entinostat decreased the mitosis‐promoting cyclin B1. Moreover, the antiproliferative cyclin‐dependent kinase inhibitor (CDKi) p16 accumulated p53 independently in response to entinostat (Fig. 1F). Another CDKi, p19, was unaffected by these treatments. Irinotecan alone and combined with entinostat decreased the phosphorylation of histone H3 at serine 10, a G2/M maker, in both genotypes. These data demonstrate that irinotecan activates the G2/M checkpoint p53 independently.

The accumulation of p16 after entinostat (Fig. 1F) and cell cycle arrest after irinotecan treatment (Fig. 1D,E) did not evoke senescence after 24 h (Fig. S1E). Few senescent (β‐galactosidase‐positive) cells appeared in HCT116^wt^ cells after a 48 h treatment with entinostat and more prominently upon cotreatment with irinotecan. However, most cells remained β‐galactosidase‐negative (Fig. S1F).

Organoids from primary CRC tumors and xenotransplantation models represent predictive preclinical models [39]. Interestingly, organoids from p53 wild‐type CRC and adjacent nonmalignant tissue revealed that irinotecan plus entinostat significantly eliminated tumor organoids but spared healthy tissue organoids (*P* < 0.001 for treated tumor organoids to control and to healthy organoids) (Fig. 1G,H). Using a BALB/c cell transformation assay [32], we further noted that this drug combination reduced the number of malignancy‐associated type‐III foci significantly (*P* < 0.01) (Fig. S1G).

Transplantation of HROC24 cells into mice demonstrated that irinotecan plus entinostat significantly suppressed the growth of established CRC tumors (*P* < 0.001 tumor volume; *P* < 0.01 tumor weight) (Fig. 1I–K). This was not associated with a weight loss of mice, and we observed no general toxicity judging liver weights (Fig. S1H,I).

These data show that irinotecan plus entinostat significantly impairs the growth and survival of p53‐positive CRC cells, transformed cell foci, tumor organoids, and established tumors in mice.

### 3.2 Irinotecan plus entinostat causes DNA damage and attenuates HR

**Fig. 2:**
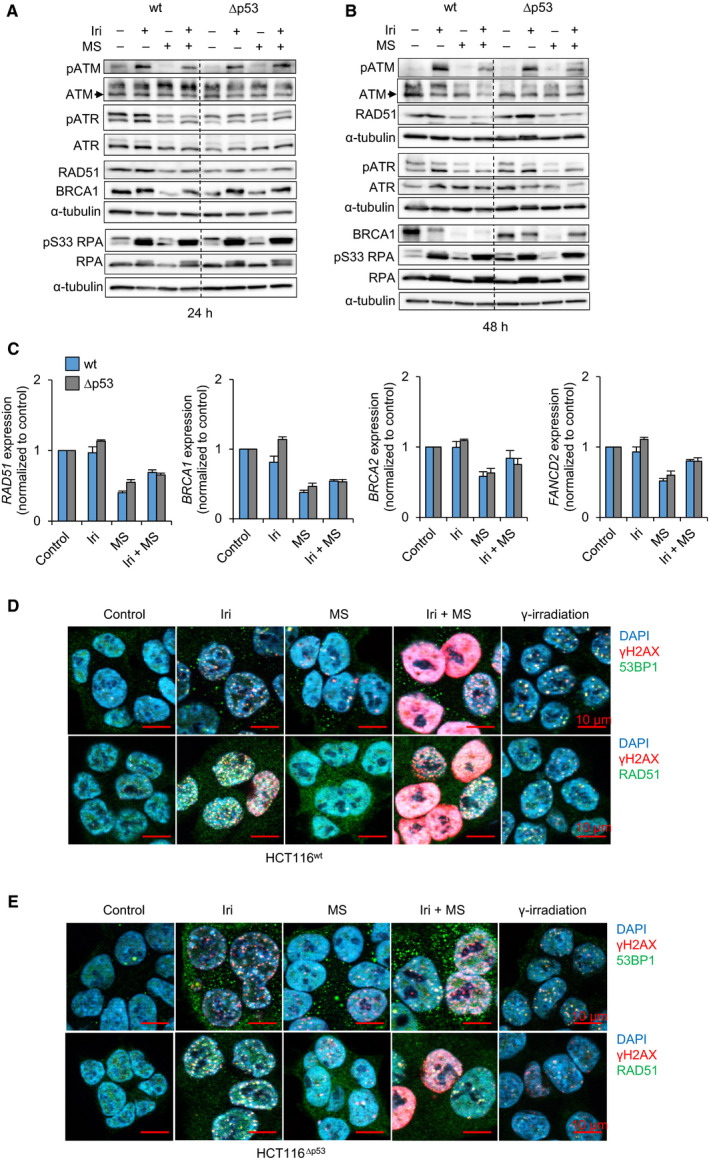
Impaired homologous repair after irinotecan plus entinostat combination in HCT116^wt^ cells. HCT116^wt^ and HCT116^Δp53^ cells were exposed to 10 µm irinotecan (Iri) ± 2 µm entinostat (MS, short for MS‐275). Expression levels of indicated proteins were analyzed by immunoblot after 24 h (A) and 48 h (B). α‐tubulin was used to control protein loading. The mRNA expressions of indicated genes (C) were quantified by qRT‐PCR after 24 h. HCT116^wt^ (D) and HCT116^Δp53^ (E) cells were exposed to 10 µm Iri ± 2 µm MS for 24 h or irradiated with 2 Gy for 2 h before fixation. The expression and localization of 53BP1 (green; upper panels) or RAD51 (green; lower panels) together with γH2AX (red) was analyzed by fluorescence microscopy. DAPI was used as a nuclear stain. Scale bars are equal to 10 µm. All graphs show the mean value of independent experiments ± SEM; immunoblots and microscopy pictures are representative of three independent experiments.

Since HR is the main repair pathway that prevents lethal DNA damage upon TOP1 inhibition [21, 22, 40], we considered that apoptosis was associated with a dysregulation of HR proteins by irinotecan plus entinostat. We investigated such proteins and found that irinotecan induced the phosphorylation of the checkpoint kinases ATM (pATM) and ATR (pATR), of RPA (pRPA), and increased the levels of BRCA1 and RAD51. Entinostat decreased BRCA1 and RAD51 below basal levels and attenuated pATR and pATM in response to irinotecan, with stronger effects in HCT116^wt^ cells than in HCT116^Δp53^ cells (Fig. 2A). After 48 h, these inhibitory effects of entinostat on HR proteins became more obvious (Fig. 2B). Contrary to HCT116^wt^ cells, HCT116^Δp53^ cells maintained ATM and BRCA1. Entinostat decreased the mRNAs of *RAD51, BRCA1/BRCA2*, and *FANCD2* in HCT116^wt^ and HCT116^Δp53^ cells (Fig. 2C).

Checkpoint kinases catalyze the phosphorylation of histone H2AX (γH2AX) [21]. Immunofluorescence for γH2AX foci and their colocalization with the DNA repair proteins 53BP1 (indicating DNA double‐strand breaks) or RAD51 (indicating HR foci) revealed that irinotecan induced foci of 53BP1/RAD51 with γH2AX comparably in HCT116^wt^ and HCT116^Δp53^ cells (Fig. 2D,E, Figs S2A,B and S3; γ‐irradiation as positive control). Entinostat slightly induced γH2AX foci (Fig. 2D,E). Irinotecan plus entinostat evoked pan‐γH2AX staining in HCT116^wt^ cells, a sign of apoptosis [41], and reduced the number of γH2AX foci that colocalized with 53BP1/RAD51 (Fig. 2D, Fig. S2A,B). HCT116^Δp53^ cells still had these foci after such treatment and the number of pan‐γH2AX‐stained cells was smaller than in HCT116^wt^ cells (Fig. 2E, Fig. S3). As in HCT116^wt^ cells, γH2AX accumulated in HROC24 cells (Fig. S2C).

We conclude that irinotecan plus entinostat evokes significantly more DNA lesions in p53‐proficient than in p53‐negative CRC cells.

### 3.3 Irinotecan plus entinostat modulates p53 PTMs

**Fig. 3:**
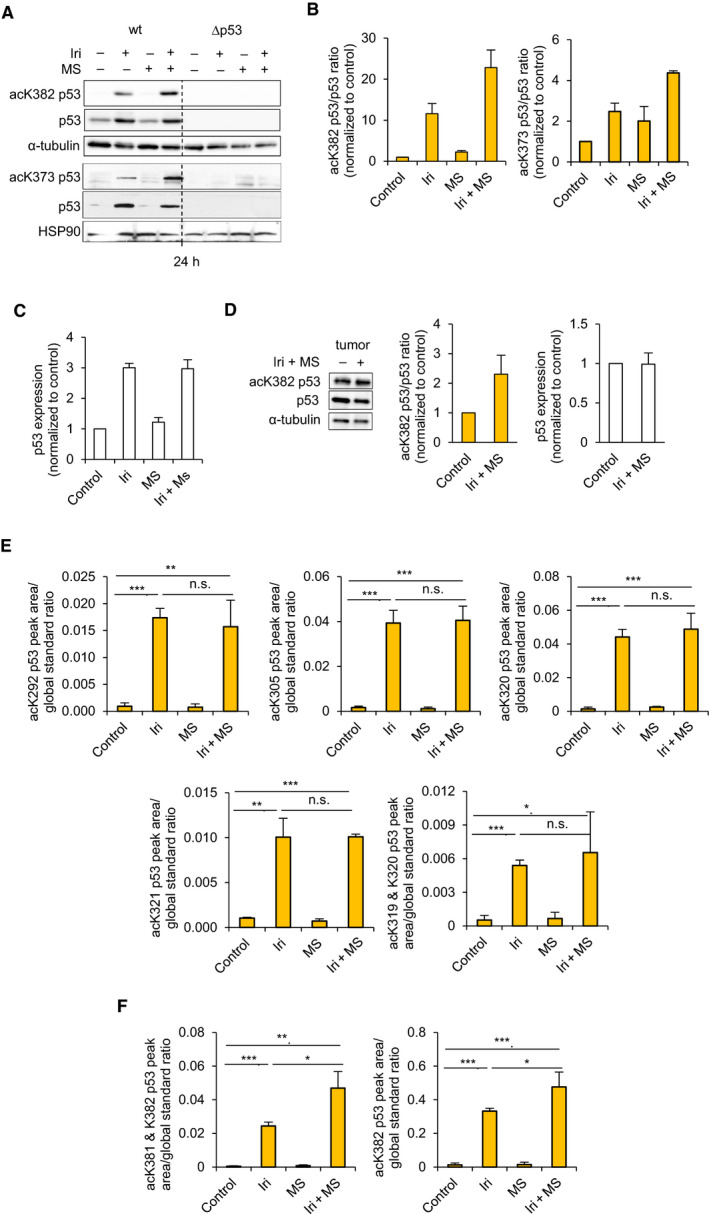
The irinotecan/entinostat cotreatment induces specific p53 acetylation patterns and apoptosis. HCT116^wt^ and HCT116^Δp53^ cells were exposed to 10 µm irinotecan (Iri) ± 2 µm entinostat (MS, short for MS‐275) for 24 h. (A) Levels of indicated proteins and acetylation of p53 were analyzed by immunoblot after 24 h. HSP90 and α‐tubulin are loading controls. Quantification of p53 acetylation was done by imagej; the ratios of acetylated over total p53 for lysine 373 (acK373) and 382 (acK382) (B) were calculated. Quantification of total p53 level is shown in (C). (D) Levels of p53 acetylation in human tumor organoid cultures were analyzed by immunoblot after 24‐h treatment. Quantification of corresponding p53 acetylation and total p53 level was done by imagej. Acetylation of lysine residues 292 (acK292), 305 (acK305), 320 (acK320), 321 (acK321), 319, and 320 (acK319 & acK320) (E) as well as acetylation of lysine moieties 381 and 382 (acK381 and acK382) and 382 (acK382) (F) were detected in different protein fragments of p53 by targeted liquid chromatography electron spray ionization mass spectrometry (LC‐ESI‐MS). Levels of each modification were shown relative to a global standard (p53 tryptic peptide 343–351). (A, B, D) show the mean value of two independent experiments ± SEM. (C, E, F) show the mean value of four independent experiments ± SEM. Significances for this figure are: *P < 0.05; **P < 0.01; ***P < 0.001 using two‐tailed *t*‐tests.

Since PTMs critically control p53 [13, 17], we analyzed their regulation by irinotecan plus entinostat. Irinotecan strongly induced p53 protein expression (threefold) and acetylation of its lysine residues 373 (acK373; 2.5‐fold), 382 (acK382; 11.6‐fold), 381 (acK381, 1.9‐fold), and 305 (acK305, 4.5‐fold). Entinostat increased the acetalytion of p53, but did not change p53 levels (Fig. 3A–C, Fig. S4A,B). Cotreatment of cells with both agents further increased C‐terminal acK373‐, acK381‐, and acK382‐p53 (4.4‐fold; 3.2‐fold; and 22.8‐fold); total p53 and acK305‐p53 were expressed as in irinotecan‐treated cells.

p53 was also strongly acetylated at K382 in CRC organoids (2.3‐fold), RKO, HROC24, and HHC6548 CRC cells after treatment with irinotecan plus entinostat (Fig. 3D, Fig. S4C,D). This finding indicates a general induction of this PTM by this drug combination.

To obtain a comprehensive view on p53 acetylation, we analyzed p53 immunoprecipitates by mass spectrometry (Fig. 3E,F). Analysis of tryptic peptides from purified p53 disclosed that irinotecan induced the acetylation of K292, K305, K319/320, K320, K321, K381/382, and K382. Irinotecan plus entinostat particularly increased the acetylation of the C‐terminal K381/382 and K382 residues (*P* < 0.05 to irinotecan) (Fig. 3F), with acK382 being the most abundant PTM. Therefore, we used acK382 as general but not exclusive marker for p53 C‐terminal acetylation in the following experiments.

### 3.4 Entinostat modulates p53 and BCL2 family members

**Fig. 4:**
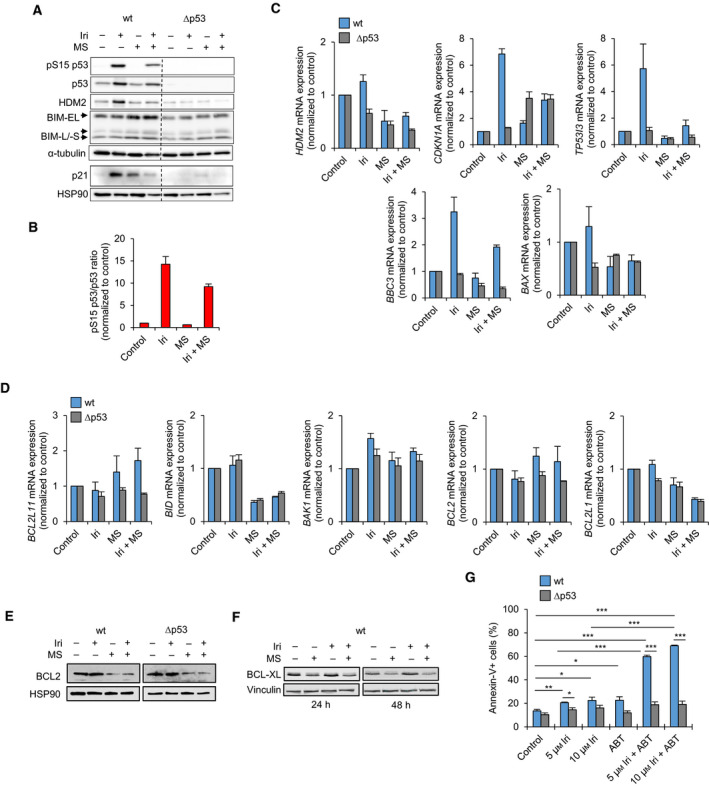
Entinostat alters the expression of BCL2 family members to promote apoptosis. HCT116^wt^ and HCT116^Δp53^ cells were exposed to 10 µm irinotecan (Iri) ± 2 µm entinostat (MS, short for MS‐275). Levels of indicated proteins and phosphorylation of p53 were analyzed by immunoblot after 24 h. HSP90 and α‐tubulin are loading controls. Quantification of p53 phosphorylation was done by imagej; the ratio of phosphorylated (pS15‐p53) over total p53 (B) was calculated. (C) The mRNA expression of indicated genes were quantified by qRT‐PCR after 24 h. (D) Expression level of BCL2 was analyzed by immunoblot after 24 h. HSP90 served as loading control. HCT116^wt^ cells were exposed to 5 µm Iri ± 2 µm MS. (E) Expression level of BCL‐XL was analyzed by immunoblot after 24 and 48 h. Vinculin served as loading controls. HCT116^wt^ and HCT116^Δp53^ cells were exposed to 5–10 µm Iri ± 500 nm ABT‐263 (ABT; navitoclax) for 48 h. The percentage of apoptotic, annexin‐V‐positive cells was quantified by flow cytometry afterward. (A–F) show the mean value of two independent experiments ± SEM. (G) shows the mean value of three independent experiments ± SEM. Significances for this figure are: *P < 0.05; **P < 0.01; ***P < 0.001 using two‐tailed *t*‐tests.

N‐terminal phosphorylation of p53 regulates its transcriptional activity [13, 17]. Irinotecan induced p53 phosphorylation at serine 15 (pS15; 14.3‐fold) (Fig. 4A). Contrary to the increased C‐terminal acetylation, entinostat decreased pS15‐p53 (9.2‐fold) in irinotecan‐treated HCT116^wt^ cells (Fig. 4A,B). This made us analyze the relationship between PTMs and targets of p53. Irinotecan induced the protein levels of p21 and HDM2 in HCT116^wt^ but not in HCT116^Δp53^ cells. This was abrogated by entinostat (Fig. 4A, Fig. S4E).

Congruently, irinotecan elevated the mRNA expression of the p53 target genes *BAX* (1.3‐fold), *HDM2* (1.3‐fold), *CDKN1A* (p21; 6.9‐fold), *TP53I3* (PIG‐3; 5.7‐fold), and *BBC3* (PUMA; 3.3‐fold). Entinostat reduced these effects (Fig. 4C). None of these genes were induced in HCT116^Δp53^ cells, except for *CDKN1A*. p21 was though barely detectable by immunoblot in such cells (Fig. 4A, Fig. S4E).

We further analyzed the mRNA levels of *BCL2L11* (BIM), *BAK1*, *BCL2*, *BCL2L1* (BCL‐XL), and *BID*, which are apoptosis regulators of the BCL2 family [14]. *BID*, *BCL2L1* mRNAs, and the BCL‐XL protein were unaffected by irinotecan (Fig. 4D,F). Entinostat and irinotecan plus entinostat increased *BCL2, BCL2L11* mRNA, and BIM protein levels particularly in HCT116^wt^ cells (Fig. 4A,D, Fig. S4E). Entinostat alone and in combination with irinotecan reduced *BCL2L1* and *BID* mRNAs, and BCL2 and BCL‐XL proteins in both HCT116 cell lines (Fig. 4D–F). *BAK1* was slightly induced by all treatments in both genotypes (Fig. 4D). Thus, entinostat induced fundamental changes of BCL2 proteins too many ‐ a p53‐independent decrease in BCL2 and BCL‐XL and a p53‐dependent increase in BIM.

To study whether the reduction in antiapoptotic BCL2 proteins by entinostat is a mechanism for apoptosis induction, we combined the BCL2/BCL‐XL/BCL‐W inhibitor navitoclax (ABT‐263) [19] with irinotecan. Navitoclax and irinotecan alone moderately inceased the amount of apoptotic HCT116^wt^ cells (Fig. 4G). Irinotecan plus navitoclax significantly induced apoptosis in HCT116^wt^ but not in HCT116^Δp53^ cells (60–69% for 5–10 µm irinotecan plus navitoclax; each *P* < 0.001 to irinotecan and to Δp53 cells). We could reproduce this finding in HROC24 cells (Fig. S4F). As expected for a nondegrading inhibitor [19], the proapoptotic effects of irinotecan plus navitoclax were not linked to a loss of BCL‐XL (Fig. S4G).

These data illustrate that irinotecan activates the transcriptional activity of p53. Entinostat antagonizes this effect and decreases BCL2 protein levels. An inhibition of antiapoptotic BCL2 proteins in cells with RS induces apoptosis p53‐dependetly.

### 3.5 Acetylated p53 accumulates at mitochondria after application of irinotecan and entinostat

**Fig. 5:**
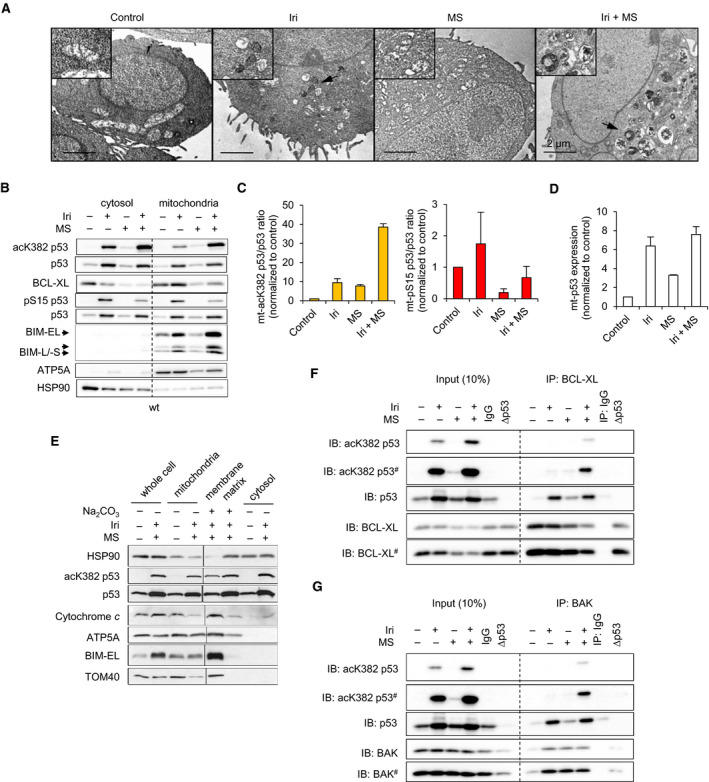
Irinotecan plus entinostat induces C‐terminal acetylation of p53 and mitochondrial damage. HCT116^wt^ cells were exposed to 10 µm irinotecan (Iri) ± 2 µm entinostat (MS, short for MS‐275) for 24 h. (A) Mitochondrial morphologies were analyzed by TEM. Representative mitochondria are shown enlarged in the top left corner of each panel. Black arrows indicate damaged mitochondria. Scale bars are equal to 2 µm. (B) Cell lysates were fractionated into mitochondria and cytosol. The expression of indicated proteins, localization, and modification of p53 was analyzed by immunoblot. HSP90 and ATP5A (ETC complex V) served as protein loading control within individual compartments. Quantification of corresponding p53 PTMs was done by imagej; the ratios of mitochondrial (mt) pS15‐ and mt‐acK382‐ over total mt‐p53 (C) were calculated. Quantification of total mt‐p53 level is shown in (D). (E) Mitochondria were further fractionated into their membranes and matrixes by NaCO_3_ treatment. The expression and localization of indicated proteins and acetylation of p53 were analyzed by immunoblot. HSP90, TOM40, and ATP5A served as loading controls for the compartments. BCL‐XL (F) and BAK (G) were immunoprecipitated from cell lysates, and expression levels of indicated proteins and acetylation of p53 were analyzed by immunoblot. (A–G) are representative/show the mean value of two independent experiments ± SEM; # indicates long exposure of the immunoblots shown in (F, G).

Since there is a loss of ΔΨ_M_ in p53‐proficient cells in response to a treatment with irinotecan and entinostat (Fig. 1A,B), we analyzed mitochondrial morphology by transmission electron microscopy (TEM) (Fig. 5A, Fig. S5A). HCT116^wt^ cells had bean‐shaped mitochondria with intact *cristae*. After irinotecan treatment, mitochondria were smaller and spherical. Irinotecan plus entinostat increased such mitochondrial damage and degradation (Fig. 5A). HCT116^Δp53^ cells showed less severe mitochondrial defects (Fig. S5A), confirming ΔΨ_M_ measurements (Fig. 1A).

Transmission electron microscopy and mitochondrial fractionation followed by immunoblot revealed that p53 was present at mitochondria in untreated cells (Fig. 5B–D, Fig. S5B). Irinotecan caused a strong accumulation of p53, pS15‐p53, and acK382‐p53 in both mitochondrial and cytosolic compartments. Irinotecan plus entinostat increased acK382‐p53 and decreased pS15‐p53 levels without an effect on total p53 (Fig. 5B–D). Intriguingly, acK382‐p53 accumulated more in mitochondrial than in cytosolic fractions when we applied irinotecan plus entinostat (Fig. 5B). This suggests that acK382‐p53 prominently locates to and interacts with proteins at mitochondria. Similar observations in RKO and HROC113 cells (Fig. S5C–H) show a general accumulation of mitochondrial (mt)‐acK382‐p53 and depletion of mt‐pS15‐p53 in CRC cells that are treated with irinotecan plus entinostat. We further found that p53 and acK382‐p53 localized at the mitochondrial membrane and matrix after this treatment (Fig. 5E).

Moreover, irinotecan increased the levels of mitochondrial BCL‐XL and BIM. Addition of entinostat to HCT116^wt^, RKO, and HROC113 cells that were cotreated with irinotecan decreased BCL‐XL and BAX but further increased BIM (Fig. 5B, Fig. S5C,F,I). Moreover, BIM accumulated at mitochondrial membranes and cytochrome *c* was released from the mitochondrial intermembrane space into the cytosol after irinotecan plus entinostat (Fig. 5E), indicating MOMP [14].

K120 acetylation of p53 promotes its interactions with HDM2, BCL2 proteins, and apoptosis induction upon DNA damage [16, 25]. Entinostat and camptothecin (irinotecan is the clinically approved derivative thereof) induce acK120‐p53 in lung and breast tumor cells [16]. In our study, acK120 escaped detection by mass spectrometry. To still understand the role of acK120‐p53, we used human p53‐negative H1299 non‐small‐cell lung cancer cells with a tetracycline‐inducible expression of p53^wt^ or p53^K120R^, which cannot be acetylated at K120 [25]. Both p53^wt^ and p53^K120R^ translocated to mitochondria after treatment with irinotecan alone or with entinostat. However, acK382‐p53 was only induced by irinotecan plus entinostat (Fig. S5J–K). ΔΨ_M_ loss was higher in p53^wt^ than in p53^K120R^ and noninduced H1299 cells after irinotecan treatment. While irinotecan plus entinostat induced a comparable ΔΨ_M_ loss in H1299 cells with p53^wt^ and p53^K120R^ (86.6% and 66.9%); noninduced H1299 cells were even less affected than with irinotecan (27.5%) (Fig. S5L), suggesting that the acetylation of K120 is not required for the synergistic apoptosis induction by irinotecan and entinostat.

This finding encouraged us to further analyze the C‐terminal acetylation of p53. Since Entinostat attenuated p53 target gene expression upon irinotecan‐induced RS (Fig. 4), we hypothesized that the C‐terminal acetylation of p53 promoted its interaction with BCL2 proteins. The activation and oligomerization of BAK by p53 can induce MOMP [14, 20]. Therefore, we immunoprecipitated BAK, as well as BCL‐XL, which binds and inactivates proapoptotic BCL2 proteins [14]. Irinotecan alone and in combination with entinostat induced clearly and specifically detectable p53‐BAK and p53‐BCL‐XL complexes (Fig. 5F,G). Entinostat alone just slightly induced these protein–protein interactions. Notably, p53 in the p53‐BAK and p53‐BCL‐XL complexes was acetylated at K382 only in cells that were treated with both irinotecan and MS‐275.

We deduce that C‐terminally acetylated p53 and BIM accumulate at mitochondria and that acK382‐p53 binds to BAK and BCL‐XL in cells that are incubated with irinotecan plus entinostat.

### 3.6 C‐terminal acetylation of p53 by CBP/p300 is key for apoptosis induction during RS

The results above suggest that C‐terminal acetylation of p53 triggers MOMP. The HATs CBP and p300 acetylate and HDAC1,‐2,‐3 deacetylate the C terminus of p53 [13, 17]. Using the CellMiner online platform [37], we investigated the expression patterns of these enzymes in 60 cancer cell lines of various origins (Tables [Link], [Link]). We noted lower mRNA transcript levels of *CREBBP* (encodes CBP) and more frequently of *EP300* (encodes p300) (*z*‐value < 0) and higher transcript levels of *HDAC1, HDAC2, and HDAC3* mRNAs in CRC cells (Table S2). Moreover, CRC cells have particularly high mutation rates in *CREBBP* and *EP300* genes when compared to other cancer cell types (Table S3).

Analyzing The Cancer Genome Atlas (TCGA) [38], we found significantly lower *CREBBP* and *EP300* expression in tumors of patients with colon adenocarcinoma (COAD) and rectum adenocarcinoma (READ) compared with matched nonmalignant tissues (Fig. S6A,B). Again, *HDAC1,‐2,‐3* mRNA transcripts were significantly overexpressed in COAD and READ samples (Fig. S6C–E). Concerning BCL2 family genes, we found significantly lower levels of *BCL2* and *BCL2L11* and significantly higher *BCL2L1* expression in COAD and READ than within matched nonmalignant tissues (Fig. S7), suggesting physiologically relevant functions of BCL‐XL and BIM in CRC tumors.

**Fig. 6:**
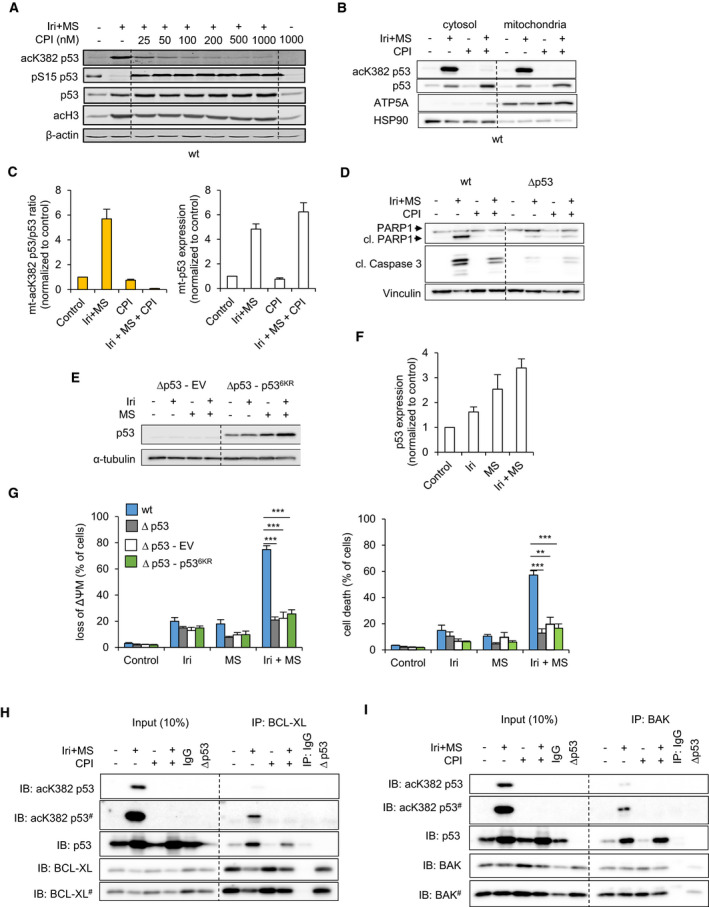
CBP/p300 activity is necessary to acetylate the C terminus of p53 and to induce apoptosis. HCT116^wt^ were exposed to 10 µm irinotecan (Iri) + 2 µm MS‐275 (MS) ± 25–1000 nm CPI‐1612 (CPI) for 24 h. (A) Levels of indicated proteins and PTMs of p53 were analyzed by immunoblot. β‐actin served as loading control. HCT116^wt^ and HCT116^Δp53^ cells were exposed to 10 µm Iri + 2 µm MS ± 200 nm CPI‐1612. (B, C) Cell lysates were fractionated into mitochondria and cytosol after 24 h. The expression of indicated proteins, localization, and acetylation of p53 was analyzed by immunoblot (B). HSP90 and ATP5A served as protein loading controls within individual compartments. Quantification of p53 acetylation was done by imagej; the ratios of mitochondrial (mt) acK382‐ over total mt‐p53 (C) were calculated, and the expression of total mt‐p53 is shown. (D) Levels of indicated proteins were analyzed by immunoblot after 48 h. Vinculin served as loading control. Stably transfected HCT116^Δp53^ cells expressing either empty vector (Δp53‐EV) for control or an acetylation incompetent p53^6KR^ mutant protein (Δp53‐p53^6KR^) were exposed to 10 µm Iri ± 2 µm MS. (E) Levels of p53 were analyzed by immunoblot after 24 h and quantified by imagej and are shown in (F); α‐tubulin served as loading control. HCT116^wt^, HCT116^Δp53^, and stably transfected HCT116^Δp53^ expressing either EV or p53^6KR^ were exposed to 10 µm Iri ± 2 µm MS. (G) The loss of ΔΨ_M_ and cell death were quantified by flow cytometry after 48 h. HCT116^wt^ and HCT116^Δp53^ cells were exposed to 10 µm Iri + 2 µm MS ± 200 nm CPI‐1612. BCL‐XL (H) and BAK (I) were immunoprecipitated from cell lysates, and expression levels of indicated proteins and acetylation of p53 were analyzed by immunoblot afterward. (A–F) and (H, I) are representative/show the mean value of two independent experiments ± SEM. # indicates long exposure of the immunoblots shown in (H, I). (G) shows the mean values of four independent experiments ± SEM. Significances for this figure are: *P < 0.05; **P < 0.01; ***P < 0.001 using two‐tailed *t*‐tests.

The low abundance of CBP/p300 transcripts, their high degree of mutations, and the high expression of HDACs targeting the p53 C terminus in CRC suggests that such cells require low levels of acetylated p53 for survival. Therefore, we analyzed the relevance of CBP/p300 with their novel inhibitor CPI‐1612 [24]. In cells treated with irinotecan plus entinostat, CPI‐1612 dose dependently suppressed the acetylation of p53 at K382 and did not reduce total p53, pS15‐p53, or histone H3 acetylation (Fig. 6A). These data verify the specificity of CPI‐1612, which equally suppressed the accumulation of acK382‐p53 in irinotecan plus entinostat‐treated RKO, HROC24, and HHC6548 CRC cells (Fig. S8A,B). Likewise, CPI‐1612 abolished acK382‐p53 levels in cytosolic and mitochondrial fractionations of HCT116^wt^, RKO, HROC24, and HROC113 cells (Fig. 6B,C, Fig. S8C–H). The stabilization and mitochondrial accumulation of p53 in response to irinotecan plus entinostat were though not reduced by CPI‐1612.

Functional analyses demonstrated that CPI‐1612 prevented the cleavage of PARP1 and reduced caspase‐3 activation in HCT116^wt^ but not in HCT116^Δp53^ cells that were treated with irinotecan plus entinostat (Fig. 6D). CPI‐1612 also suppressed apoptosis induction by this drug combination in HROC24 cells (Fig. S8I) and CPI‐1612 significantly lowered the number of apoptotic cells in HCT116^wt^ and RKO cells that were treated with irinotecan plus navitoclax (Fig. S8J,K). These data show that CPI‐1612 has an antiapoptotic effect in CRC cells expressing p53 and that the C‐terminal acetylation of p53 is crucial to trigger apoptosis.

To complement these data, we stably reconstituted HCT116^Δp53^ cells with p53^6KR^, which cannot be acetylated at its C terminus (Fig. S8L). p53^6KR^ accumulated in response to irinotecan or entinostat, with strongest effects seen with their combination (Fig. 6E,F). Measurement of ΔΨ_M_ loss revealed that p53^6KR^ did not sensitize cells to irinotecan plus entinostat (Fig. 6G) and CPI‐1612 did not reduce the levels of cleaved PARP1 and caspase‐3 in stable p53^6KR^ and EV‐transfected HCT116 cells that were treated with these agents (Fig. S8M). Nonetheless, p53^6KR^ accumulated at mitochondria after treatment (Fig. S8L). Hence, C‐terminal acetylation of p53 is not essential for its mitochondrial accumulation but for apoptosis induction.

As CPI‐1612 suppressed apoptosis induction by irinotecan plus entinostat or navitoclax, we speculated that C‐terminally acetylated p53 interacts with regulators of mitochondrial apoptosis. Again, we immunoprecipitated BAK and BCL‐XL and tested for bound p53 and acK382‐p53. CPI‐1612 abolished the C‐terminal acetylation of p53, and consequently, the presence of acK382‐p53 in BAK and in BCL‐XL complexes in HCT116 cells (Fig. 6H,I). Total p53 was though still bound to BAK or BCL‐XL. These observation were confirmed in HROC24 and RKO cells (Fig. S9), illustrating a general mechanism in CRC cells and the necessity of p53‐BAK and p53‐BCL‐XL complexes containing C‐terminally acetylated p53 for MOMP‐driven apoptosis.

These data define a novel acetylation‐dependent pathway of p53‐mediated mitochondrial apoptosis in CRC cells. The modulation of antiapoptotic BCL2 proteins by entinostat does not kill CRC cells with RS in the absence of C‐terminally acetylated p53, which binds BAK and BCL‐XL. Hence, this acetylation of p53 is a key trigger for apoptosis upon RS and HDAC inhibition. This notion extends the perspective on how p53 initiates apoptosis, since until now its accumulation at mitochondria and acetylation within its DBD are believed to trigger MOMP [12, 15, 20].

## 4 Discussion

Late‐stage CRC requires novel therapies [42]. We show that combinations of irinotecan plus entinostat efficiently kill p53‐positive CRC cells that grow *in vitro* and *in vivo* as well as malignant cell foci formation and CRC tumor organoids, illustrating translational relevance. Moreover, our data suggest that class I HDAC inhibition allows reducing the doses of irinotecan that need to be given to patients. This is of clinical relevance because irinotecan is a frequently applied drug that, unfortunately, produces severe side effects that lead to a discontinuation of its use [21, 23].

We observed that irinotecan induced various PTMs of p53, including the acetylation of several lysine residues and N‐terminal phosphorylation. In parallel, we found elevated p53 target gene expression, activated caspase‐3, and cleaved PARP1 in irinotecan‐treated HCT116^wt^ cells. Entinostat specifically increased the irinotecan‐induced acetylation of the C‐terminal p53 residues K373, K381, and K382 in CRC cells and organoids with strongest effects on K382. Other acetylation sites of p53 in the tetramerization domain (K292, K305, K320) were not altered upon the addition of entinostat. While this does not imply that acetylated K373 and K381 are less important for apoptosis induction than acetylated K382, we can connect increased mitochondrial acK382‐p53 levels to MOMP induction and apoptosis of cells that are treated with combinations of irinotecan plus entinostat. A role of C‐terminally acetylated p53 and its relevance for irinotecan‐induced apoptosis have not been described before. Such insights are relevant in light of the sparsely defined molecular markers for therapeutic effects of HDACi alone and when combined with standard chemotherapeutics [2, 7, 43]. Apparently, the presence of C‐terminally acetylated p53 promotes the proapoptotic activity of BAK and impairs the antiapoptotic functions of BCL‐XL a and CBP/p300 and HDAC1,‐2,‐3 are antagonistic gatekeepers of this process.

We further demonstrate that the acetylation of the p53 C terminus does not significantly alter the mitochondrial localization of p53 and its interaction with BAK and BCL‐XL. These data are coherent to the current literature which reports that the binding of these proteins is mediated through the p53 DBD [16, 17, 20, 44, 45, 46, 47]. Nonetheless, the presence of acK382‐p53 in BAK and BCL‐XL complexes appears decisive for whether irinotecan plus entinostat induces apoptosis. This finding confirms the notion that p53‐BAK and p53‐BCL‐XL complexes trigger apoptosis [12, 13, 20] and we present a pharmacologically feasible way to enhance the apoptotic activity of this complex. The acetylation of p53 at K120 (within its DBD) does not determine its interaction with BCL‐XL and does not activate BAK directly but replaces it from MCL‐1 to initiate apoptosis [16, 20, 25]. In accordance herewith, we show that K120 is important for p53's functions after irinotecan treatment. However, it is not essential for the mitochondrial translocation of p53, its stability, and MOMP induction after irinotecan plus entinostat treatment. We provide evidence that the C‐terminally acetylated p53 which binds BAK and BCL‐XL is decisive for apoptosis induction. Congruent herewith, p53^6KR^ cannot enhance apoptosis in irinotecan plus entinostat‐treated cells compared with irinotecan, although these drugs stabilize p53^6KR^.

We further discriminate how the acetylation of p53 in cells with RS affects its ability to induce apoptosis as transcription factor. Although the C‐terminal acetylation can increase the promotor binding specificity of p53 [48, 49], entinostat lowered the irinotecan‐induced N‐terminal phosphorylation of p53 and the induction of its target genes in our systems. These lower pS15‐p53 levels might be explained by a reduced activation of the checkpoint kinases ATM and ATR, which phosphorylate p53 [8, 13, 17], in entinostat‐treated cells. Recent data consistently demonstrate that the acetylation of p53 is more critical than its nuclear abundance, promoter binding, and stability [47, 50, 51].

Our finding that the CBP/p300 inhibitor CPI‐1612 suppresses C‐terminal acetylation of p53 in irinotecan plus entinostat‐treated CRC cells and consequently apoptosis supports our model of a proapoptotic switch that depends on C‐terminal acetylation of p53. We could confirm the specificity of CPI‐1612 by a lack of effect on histone acetylation, which is catalyzed by several HATs [1, 6]. Furthermore, we present evidence that p53 is critical for the biological effects of CPI‐1612. This agent strongly attenuated apoptosis induction by irinotecan plus entinostat in HCT116^wt^, HROC24, and RKO but not in HCT116^Δp53^ and p53^6KR^‐reconstituted cells, excluding p53‐independent antiapoptotic effects.

Irinotecan evokes RS and lethal DNA damage upon prolonged replication fork stalling and if DNA lesions are not repaired [21]. Our applied concentration range of irinotecan alone had very similar, moderate cytotoxic effects on HCT116^wt^ and HCT116^Δp53^ cells, indicating that such apoptosis induction can occur even in the absence of p53. Others likewise noted that p53‐positive fibroblasts and glioma cells are even less sensitive to DNA damage and apoptosis induction by irinotecan. These authors suggested that a p53‐dependent proteasomal degradation of the TOP1 complex on DNA prevents that stalled replication forks turn into proapoptotic DNA strand breaks [52]. HCT116^wt^ and HCT116^Δp53^ cells respond also very similarly to entinostat [29]. We noted a p53‐independent accumulation of HR proteins, including RAD51 and that proapoptotic effects of irinotecan plus entinostat in p53‐positive cells corelate with low HR protein and mRNA levels. This occurs more strongly in HCT116^wt^ than in HCT116^Δp53^ cells, suggesting a suppression of DNA repair by acetylated p53. Entinostat decreased the irinotecan‐induced RAD51 in irinotecan treated cells and 53BP1 foci in HCT116^wt^ cells and induced apoptosis‐associated pan‐nuclear γH2AX. In p53‐negative cells, RAD51 and 53BP1 foci remained present after irinotecan plus entinostat and cells carrying pan‐nuclear γH2AX were low. This indicates a link between functional HR and suppression of apoptosis. The early impact of p53 on RAD51 protein expression appears to support the apoptosis induction after the irinotecan‐induced RS and DNA damage. This might also apply to the loss of BRCA1, full‐length PARP1, and the attenuation of pATM/pATR. The latter might be due to the induction of the phosphatase‐2A subunit PR130 [8]. We further show that entinostat attenuates BCL‐XL expression, probably reflecting an abrogation of the RS‐induced NF‐κB/p53 crosstalk by HDACi [53]. Entinostat additionally attenuates BCL2 similar to BCL‐XL, which both inactivate BAK [14, 20]. Hence, their reduction in company with p53‐BCL‐XL complex formation can promote p53‐mediated BAK oligomerization and MOMP induction. p53‐mediated apoptosis induction via PUMA and NOXA requires BIM, which can, similar to p53, release BAK form antiapoptotic BCL2 proteins [54, 55]. *BBC3* and *BCL2L11* mRNAs and BIM protein levels increase in HCT116^wt^ cells that are incubated with irinotecan plus entinostat but not in HCT116^Δp53^ cells. This suggest that the p53‐PUMA‐BIM‐axis contributes to apoptosis in irinotecan plus entinostat‐treated cells.

A major finding of this work is that a complex of BAK with C‐terminally acetylated p53 is a CBP/p300‐dependent, previously unknown master switch for MOMP and apoptosis. As long as HDAC1,‐2,‐3 are active, they prevent the formation of the complex containing C‐terminally acetylated p53 and BAK. Since RS and DNA damage also occur endogenously in rapidly growing cancer cells [56], such cells should be selected for low CBP/p300 and high HDAC1,‐2,‐3 expression. Are there hints for a physiological relevance for the regulation of p53 C‐terminal acetylation by class I HDACs and CBP/p300? Indeed, large‐scale analysis of tumor samples illustrates that CBP/p300 are low and that HDAC1, ‐2, ‐3 are highly expressed in primary CRC tumors and CRC cell lines. This indicates that a physiologically important function of p53 acetylation is to impair the development, growth, and survival of CRC. A dysregulation of HDACs and HATs could disengage C‐terminal acetylation of p53 as an antitumorigenic apoptotic principle.

## 5 Conclusion

From our data, we deduce that an accumulation of C‐terminally acetylated p53 at mitochondria, its direct interactions with BCL2 family proteins, and a shift of antiapoptotic toward proapoptotic BCL2 family protein/gene expression upon class I HDAC inhibition pushes cells with RS into apoptosis. Combinations of irinotecan plus entinostat drastically cause mitochondrial injury and DNA damage in p53‐positive cells. This is connected to (a) high C‐terminal acetylation and mitochondrial localization of p53, (b) acetyl‐p53‐BAK and acetyl‐p53‐BCL‐XL complexes, (c) impaired HR and DNA repair, (d) decreased transcriptional activity of p53, (e) an imbalance in BCL2 proteins, and (f) significant intrinsic apoptosis induction in p53‐positive cancer cells. Thus, combinations of irinotecan plus entinostat might be a promising therapeutic approach for primary tumors and recurrent irinotecan‐resistant tumors.

## Conflict of interest

The authors declare no conflict of interest.

## Author contributions

CM performed immunoblots, apoptosis assays, most immunoprecipitations, DNA damage analyses, reconstitution experiments, and mitochondrial analyses. JS and LM‐B performed qRT‐PCR and concentration‐response analysis; SL and ODKM performed targeted mass spectrometry of p53 PTMs and data analysis; MB, IH, and AP‐S performed cell cycle analysis, apoptosis assays, and immunoblots; SN performed primary CRC organoid culture and treatment; TW performed immunoprecipitations; MW performed TEM analysis; FBM performed the BALB transformation assay; KS did global data search on protein expression in CRC; CSM and AP‐S performed primary CRC cell culture and treatments; SK and CSM performed animal experiments; MB, ODKM, JS, RT, ML, FN, Z‐QW, and TH contributed the scientific development, discussion, and supervision of the project; CM and OHK designed the experiments, interpreted the data, supervised the project, and wrote the manuscript.

### Peer Review

The peer review history for this article is available at https://publons.com/publon/10.1002/1878‐0261.13060.

## Supporting information


**Fig. S1.** Irinotecan plus entinostat is effective against CRC cells and tolerated by mice.HCT116^wt^ (A) and HCT116^Δp53^ (B) cells were exposed to 1‐20 µM irinotecan (Iri) ± 1‐2µM entinostat (MS‐275, abbreviated as MS). Cell death and the loss of ΔΨ_M_ were quantified by flow cytometry after 48‐h treatment periods. (C) CRC short‐term cultures were incubated with 0,5‐10 µM MS and irinotecan for 48h. HROC24 cells were treated with 2 µM Iri and HROC113/HHC6548 cells with 3 µM Iri. All graphs in (C) show the average of 3 independent experiments ± SEM; significances for this figure are: * *p*<0.05; ** *p*<0.01; *** *p*<0.001 to MS‐275; ° *p*<0.05; °° *p*<0.01; °°° *p*<0.001 to irinotecan using two‐tailed *t*‐tests. HCT116^wt^ and HCT116^Δp53^ cells were exposed to 10 µM Iri ± 2 µM MS. Additionally, cells were 1h pretreated with 20 µM zVAD alone and ± Iri/MS. (D) The loss of ΔΨ_M_ and cell death were quantified by flow cytometry after 48h. Cellular senescence was analyzed by β‐galactosidase staining after 24h (E) and 48h (F). Red arrows indicate β‐galactosidase‐positive cells. (G) Representative images of a BALB/c cell transformation assay after 72h of treatment with 10 µM Iri + 2 µM MS. MCA/TPA‐transformed cell foci are Giemsa stained and appear in blue. Quantification of the number of malignancy‐associated type‐III foci/well is shown. All figures/graphs are representative/show the mean value of 3 independent experiments ± SEM; significances for these figure are: * *p*<0.05; ** *p*<0.01; *** *p*<0.001 using two‐tailed *t*‐tests. (H,I) 6‐ to 8‐week‐old NMRI‐Foxn1nu mice were treated with 20mg/kg Iri and 2,5mg/kg MS for 16 days. (H) Changes in their body weights were monitored daily. (I) The weight of whole livers was documented after 16 days of therapy and are shown as mean values ± SEM.

**Fig. S2.** Irinotecan plus entinostat induces DNA damage in p53 wild‐type CRC cells. HCT116^wt^ cells were exposed to 10 µM irinotecan (Iri) ± 2 µM entinostat/MS‐275 (MS) for 24h or irradiated with 2Gy for 2h before fixation. The expression and localization of 53BP1 (A) or RAD51 (B) together with γH2AX (red) was analyzed by fluorescence microscopy. DAPI was used as nuclear stain. Single channels as well as merged pictures are shown. Pictures are representative for 3 individual experiments. (C) HCT116^wt^ and HROC24 cells were exposed to 5 µM Iri ± 2 µM MS for 24h. Levels of indicated proteins cells were analyzed by immunoblot; HSP90 served as loading control. Immunoblots are representative for 2 independent experiments.

**Fig. S3.** Irinotecan plus entinostat induces DNA damage in p53 null CRC cells. HCT116^Δp53^ cells were exposed to 10 µM irinotecan (Iri) ± 2 µM entinostat/MS‐275 (MS) for 24h or irradiated with 2Gy for 2h before fixation. The expression and localization of 53BP1 (A) or RAD51 (B) together with γH2AX (red) was analyzed by fluorescence microscopy. DAPI was used as nuclear stain. Single channels as well as merged pictures are shown. Pictures are representative for 3 individual experiments.

**Fig. S4.** Irinotecan plus entinostat increases the C‐terminal acetylation of p53 in CRC cells. HCT116^wt^ and HCT116^Δp53^ cells were exposed to 10 µM irinotecan (Iri) ± 2 µM entinostat/MS‐275 (MS) for 24h. (A) Protein expression levels of indicated proteins and acetylation of p53 were analyzed by immunoblot and quantified using LiCor Odyssey Software; the ratios of acetylated over total p53 were calculated (B). HSP90 served as loading control. RKO (C), HROC24, and HHC6548 (D) cells were exposed to 5 µM Iri ± 2 µM MS. Protein expression levels of indicated proteins and acetylation of p53 were analyzed by immunoblot after 24‐h treatment periods. HSP90 and β‐actin served as loading control. HCT116^wt^ and HCT116^Δp53^ cells were exposed to 10 µM Iri ± 2 µM MS. (E) Levels of indicated proteins were analyzed by immunoblot after 48h; α‐tubulin served as loading control. HCT116^wt^ and HROC24 cells were exposed to 5 µM Iri ± 500nM navitoclax (ABT‐263, abbreviated as ABT). (F) The percentage of annexin‐V‐positive (i.e., apoptotic) HROC24 cells was quantified by flow cytometry after 48h. (G) Levels of indicated proteins were analyzed by immunoblot after 24h; HSP90 served as loading control. (A‐B) show 1 representative experiment each (C‐G) are representative for/show the mean value of 2 individual experiments.

**Fig. S5.** C‐terminally acetylated p53 locates at mitochondria after treatment with irinotecan plus entinostat. HCT116^wt^ and HCT116^Δp53^ cells were exposed to 10 µM irinotecan (Iri) ± 2 µM entinostat/MS‐275 (MS) for 24h. (A) Mitochondrial morphologies in HCT116Δp53 cells were analyzed by TEM. Representative mitochondria are shown enlarged in the top left corner of each panel. Black arrows indicate damaged mitochondria. (B) Localization of immune‐gold‐labeled p53 was analyzed by TEM on freeze‐fractures from HCT116wt cells. Gold particles are indicated by black arrows and shown enlarged in the bottom left corner of each panel. RKO (C‐E) and HROC113 (F‐H) cells were exposed to 10 µM Iri ± 2 µM MS for 24h. Cell lysates were fractionated into mitochondria and cytosol. The expression of indicated proteins, localization, and modification of p53 in RKO (C) and HROC113 cells (F) was analyzed by immunoblot. ATP5A served as mitochondrial protein loading. Quantification of corresponding p53 PTMs was done by ImageJ; the ratios of mitochondrial (mt)‐pS15‐ and mt‐acK382‐ over total p53 in RKO (D) and HRCO113 cells (G), were calculated. Quantification of mitochondrial total p53 level is shown in (E) for RKO and in (H) for HROC113 cells. HCT116^wt^ and HCT116^Δp53^ cells were exposed to 10 µM Iri ± 2 µM MS for 24h. (I) Cell lysates were fractionated into mitochondria and cytosol. The expression of indicated proteins was analyzed by immunoblot. HSP90 and TOM40 served as protein loading control within individual compartments. Tetracyclin‐induced H1299 TO p53wt or H1299 TO p53K^120R^ cells were exposed to 10 µM Iri ± 2 µM MS. (J‐K) Cell lysates were fractionated into mitochondria and cytosol after 24h. The expression, localization, and acetylation of p53 was analyzed by Immunoblot. HSP90 and TOM40 served as protein loading control within individual compartments. (L) The loss of ΔΨ_M_ was quantified by flow cytometry after 48h. (A‐L) are representative for/show the mean value of 2 individual experiments ± SEM; ^#^ indicate long exposures of corresponding proteins for immunoblots.

**Fig. S6.** Differential gene expression of HATs and HDACs in human cancer samples. Gene expression of The Cancer Genome Atlas (TCGA) datasets was analyzed for indicated genes using Gene DE module in the Timer web platform. Differences in gene expression level are depicted as log2‐fold change. Colon adenocarcinomas (COAD) and rectum adenocarcinomas (READ) are highlighted. Significances for this figure are: * *p*<0.05; ** *p*<0.01; *** *p*<0.001.

**Fig. S7.** Differential gene expression of BCL2 proteins in human cancer samples. Gene expression of The Cancer Genome Atlas (TCGA) datasets was analyzed for indicated genes using Gene DE module in the Timer web platform. Differences in gene expression level are depicted as log2‐fold change. Colon adenocarcinomas (COAD) and rectum adenocarcinomas (READ) are highlighted. Significances for this figure are: * *p*<0.05; ** *p*<0.01; *** *p*<0.001.

**Fig. S8.** C‐terminally acetylated p53 is necessary to induce apoptosis by irinotecan plus entinostat. RKO (A), HROC24, and HHC6548 (B) cells were exposed to 5 µM irinotecan (Iri) + 2 µM MS‐275 (MS) ± 200nM CPI‐1612 (CPI). Levels of indicated proteins and acetylation of p53 were analyzed by immunoblot after 24h. HSP90 served as loading control. RKO (C‐D) and HROC113 (E‐F) cells were exposed to 10 µM Iri + 2 µM MS ± 200nM CPI. Cell lysates were fractionated into mitochondria and cytosol after 24h. The expression of indicated proteins, localization, and acetylation of p53 in RKO (C) and HROC113 cells (E) was analyzed by immunoblot. ATP5A served as mitochondrial protein loading. Quantification of p53 acetylation was done by ImageJ; the ratios of mitochondrial mt‐acK382‐ over total p53 in RKO (D) and HRCO113 cells (F) were calculated and the expression of total p53 is shown correspondingly. (G‐H) HROC24 cells were exposed to 10 µM Iri ± 2 µM MS ± 200nM CPI. Cell lysates were fractionated into mitochondria and cytosol after 24h. The expression of indicated proteins, localization, and acetylation of p53 was analyzed by immunoblot (G). ATP5A served as mitochondrial protein loading. Quantification of p53 acetylation was done by ImageJ; the ratios of mitochondrial mt‐acK382‐ over total p53 (H) were calculated and the expression of mitochondrial total p53 is shown correspondingly. (I) HROC24 cells were exposed to 5 µM Iri + 2 µM MS ± 200nM CPI for 48h. The percentage of apoptotic Annexin‐V‐positive was quantified by flow cytometry afterward. (J) HCT116wt cells were exposed to 5 µM Iri ± 500nM ABT‐263 (ABT) ± 200nM CPI for 48h. The percentage of apoptotic Annexin‐V‐positive was quantified by flow cytometry afterward. (K) RKO cells were exposed to 5 µM Iri + 500nM ABT ± 200nM CPI for 72h. The percentage of apoptotic Annexin‐V‐positive was quantified by flow cytometry afterward. (L) HCT116^Δ^ ^p53^ were transient transfected with empty vector (Δp53 ‐ EV) for control, wild‐type p53 (Δp53 ‐ p53wt), or acetylation incompetent p536KR (Δp53 ‐ p536KR) and were exposed to 10 µM Iri + 2 µM MS for 24h. Cell lysates were fractionated into mitochondria and cytosol. The expression of indicated proteins, localization, and acetylation of p53 was analyzed by immunoblot. HSP90 and TOM40 served as protein loading control within individual compartments. (M) Stable transfected HCT116^Δ^ ^p53^ expressing either empty vector (Δp53 ‐ EV) for control or acetylation incompetent p536KR (Δp53 ‐ p536KR) were exposed to 10 µM Iri ± 2 µM MS ± 200nM CPI. Levels of indicated proteins were analyzed by immunoblot after 48h. α‐tubulin served as loading control. (I‐J) show the mean value of 3 independent experiments ± SEM. (A‐H) and (K‐M) all immunoblots and graphs are representative for/show the mean value of 2 independent experiments ± SEM. Significances for this figure are: * *p*<0.05; ** *p*<0.01; *** *p*<0.001 using two‐tailed *t*‐tests. ^#^ indicate long exposures of corresponding proteins for immunoblots.

**Fig. S9.** C‐terminally acetylated p53 interacts with BAK to promote apoptosis. HROC24 cells were exposed to 5 µM irinotecan (Iri) ± 2 µM entinostat/MS‐275 (MS) ± 200nM CPI‐1612 (CPI). BCL‐XL (A) and BAK (B) were immunoprecipitated from cell lysates and expression levels of indicated proteins and acetylation of p53 were analyzed by immunoblot afterward. RKO cells were exposed to 10 µM Iri ± 2 µM MS ± 200nM CPI. BCL‐XL (C) and BAK (D) were immunoprecipitated from cell lysates and expression levels of indicated proteins and acetylation of p53 were analyzed by immunoblot afterward. All immunoblots and graphs are representative of 2 independent experiments. ^#^ indicate long exposures of corresponding proteins for immunoblots.

**Table S1.** Synergistic interaction of irinotecan plus entinostat combinations in CRC cells. HCT116^wt^ and HCT116^Δp53^ cells were exposed to 1‐20 µM irinotecan (Iri) ± 1‐2 µM entinostat (MS‐275, abbreviated as MS). Cell death and the loss of ΔΨ_M_ were quantified by flow cytometry after 48h treatment periods. CI‐values were calculated with CalcuSyn from these dose‐response curves (see Figures S1A‐B).

**Table S2.** Differential gene expression of HATs and HDACs in human cancer cell lines. Gene expression data of indicated genes in the NCI‐60 cell line panel were exported using the CellMiner web tool. Changes of transcript intensities are depicted as z‐scores. CRC cell lines (CO) are highlighted.

**Table S3.** Mutations of HAT and HDAC genes in human cancer cell lines. Mutations of indicated genes in the NCI‐60 cell line panel were exported using the CellMiner web tool. The number of mutations as absolute numbers. CRC cell lines (CO) are highlighted.

## Data Availability

The raw files and the MaxQuant search results files have been deposited as partial submission to the ProteomeXchange Consortium via the PRIDE partner repository [57] via the PRIDE partner repository with the dataset identifier PXD019999. Otherwise, all data generated or analyzed during this study are included in this published article (and its Supporting Information files).

## References

[1] Verdin E & Ott M (2015) 50 years of protein acetylation: from gene regulation to epigenetics, metabolism and beyond. Nat Rev Mol Cell Biol 16, 258–264. 25549891 10.1038/nrm3931

[2] Li Y & Seto E (2016) HDACs and HDAC inhibitors in cancer development and therapy. Cold Spring Harb Perspect Med 6, a026831. 27599530 10.1101/cshperspect.a026831 PMC5046688

[3] Ramaiah MJ , Tangutur AD & Manyam RR (2021) Epigenetic modulation and understanding of HDAC inhibitors in cancer therapy. Life Sci 277, 119504. 33872660 10.1016/j.lfs.2021.119504

[4] Siegel RL , Miller KD , Goding Sauer A , Fedewa SA , Butterly LF , Anderson JC , Cercek A , Smith RA & Jemal A (2020) Colorectal cancer statistics, 2020. CA Cancer J Clin 70, 145–164. 32133645 10.3322/caac.21601

[5] Singh A , Patel P , Jageshwar , Patel VK , Jain DK , Kamal M & Rajak H (2018) The safety, efficacy and therapeutic potential of histone deacetylase inhibitors with special reference to panobinostat in gastrointestinal tumors: a review of preclinical and clinical studies. Curr Cancer Drug Targets 18, 720–736. 28669336 10.2174/1568009617666170630124643

[6] Eckschlager T , Plch J , Stiborova M & Hrabeta J (2017) Histone deacetylase inhibitors as anticancer drugs. Int J Mol Sci 18, 1414. 10.3390/ijms18071414 PMC5535906 28671573

[7] Li G , Tian Y & Zhu WG (2020) The roles of histone deacetylases and their inhibitors in cancer therapy. Front Cell Dev Biol 8, 576946. 33117804 10.3389/fcell.2020.576946 PMC7552186

[8] Göder A , Emmerich C , Nikolova T , Kiweler N , Schreiber M , Kühl T , Imhof D , Christmann M , Heinzel T , Schneider G *et al*. (2018) HDAC1 and HDAC2 integrate checkpoint kinase phosphorylation and cell fate through the phosphatase‐2A subunit PR130. Nat Commun 9, 764. 29472538 10.1038/s41467-018-03096-0 PMC5823910

[9] Nikolova T , Kiweler N & Krämer OH (2017) Interstrand crosslink repair as a target for HDAC inhibition. Trends Pharmacol Sci 38, 822–836. 28687272 10.1016/j.tips.2017.05.009

[10] Meisenberg C , Ashour ME , El‐Shafie L , Liao C , Hodgson A , Pilborough A , Khurram SA , Downs JA , Ward SE & El‐Khamisy SF (2017) Epigenetic changes in histone acetylation underpin resistance to the topoisomerase I inhibitor irinotecan. Nucleic Acids Res 45, 1159–1176. 28180300 10.1093/nar/gkw1026 PMC5388393

[11] Fischer M (2017) Census and evaluation of p53 target genes. Oncogene 36, 3943–3956. 28288132 10.1038/onc.2016.502 PMC5511239

[12] Aubrey BJ , Kelly GL , Janic A , Herold MJ & Strasser A (2018) How does p53 induce apoptosis and how does this relate to p53‐mediated tumour suppression? Cell Death Differ 25, 104–113. 29149101 10.1038/cdd.2017.169 PMC5729529

[13] Hafner A , Bulyk ML , Jambhekar A & Lahav G (2019) The multiple mechanisms that regulate p53 activity and cell fate. Nat Rev Mol Cell Biol 20, 199–210. 30824861 10.1038/s41580-019-0110-x

[14] Kale J , Osterlund EJ & Andrews DW (2018) BCL‐2 family proteins: changing partners in the dance towards death. Cell Death Differ 25, 65–80. 29149100 10.1038/cdd.2017.186 PMC5729540

[15] Marchenko ND & Moll UM (2014) Mitochondrial death functions of p53. Mol Cell Oncol 1, e955995. 27308326 10.1080/23723548.2014.955995 PMC4905191

[16] Mellert HS , Stanek TJ , Sykes SM , Rauscher FJ III , Schultz DC & McMahon SB (2011) Deacetylation of the DNA‐binding domain regulates p53‐mediated apoptosis. J Biol Chem 286, 4264–4270. 21148320 10.1074/jbc.M110.184663 PMC3039376

[17] Gu B & Zhu WG (2012) Surf the post‐translational modification network of p53 regulation. Int J Biol Sci 8, 672–684. 22606048 10.7150/ijbs.4283 PMC3354625

[18] Williams AB & Schumacher B (2016) p53 in the DNA‐damage‐repair process. Cold Spring Harb Perspect Med 6, a026070. 27048304 10.1101/cshperspect.a026070 PMC4852800

[19] Mohamad Anuar NN , Nor Hisam NS , Liew SL & Ugusman A (2020) Clinical review: navitoclax as a pro‐apoptotic and anti‐fibrotic agent. Front Pharmacol 11, 564108. 33381025 10.3389/fphar.2020.564108 PMC7768911

[20] Pietsch EC , Sykes SM , McMahon SB & Murphy ME (2008) The p53 family and programmed cell death. Oncogene 27, 6507–6521. 18955976 10.1038/onc.2008.315 PMC2657599

[21] Thomas A & Pommier Y (2019) Targeting topoisomerase I in the era of precision medicine. Clin Cancer Res 25, 6581–6589. 31227499 10.1158/1078-0432.CCR-19-1089 PMC6858945

[22] Tripathi K , Mani C , Clark DW & Palle K (2016) Rad18 is required for functional interactions between FANCD2, BRCA2, and Rad51 to repair DNA topoisomerase 1‐poisons induced lesions and promote fork recovery. Oncotarget 7, 12537–12553. 26871286 10.18632/oncotarget.7247 PMC4914303

[23] Fujita K , Kubota Y , Ishida H & Sasaki Y (2015) Irinotecan, a key chemotherapeutic drug for metastatic colorectal cancer. World J Gastroenterol 21, 12234–12248. 26604633 10.3748/wjg.v21.i43.12234 PMC4649109

[24] Wilson JE , Patel G , Patel C , Brucelle F , Huhn A , Gardberg AS , Poy F , Cantone N , Bommi‐Reddy A , Sims RJ *et al*. (2020) Discovery of CPI‐1612: a potent, selective, and orally bioavailable EP300/CBP histone acetyltransferase inhibitor. ACS Med Chem Lett 11, 1324–1329. 32551019 10.1021/acsmedchemlett.0c00155 PMC7294707

[25] Sykes SM , Stanek TJ , Frank A , Murphy ME & McMahon SB (2009) Acetylation of the DNA binding domain regulates transcription‐independent apoptosis by p53. J Biol Chem 284, 20197–20205. 19494119 10.1074/jbc.M109.026096 PMC2740446

[26] Mullins CS , Micheel B , Matschos S , Leuchter M , Bürtin F , Krohn M , Hühns M , Klar E , Prall F & Linnebacher M (2019) Integrated biobanking and tumor model establishment of human colorectal carcinoma provides excellent tools for preclinical research. Cancers (Basel) 11, 1520. 10.3390/cancers11101520 PMC6826890 31601052

[27] Sato T , Stange DE , Ferrante M , Vries RGJ , van Es JH , van den Brink S , van Houdt WJ , Pronk A , van Gorp J , Siersema PD *et al*. (2011) Long‐term expansion of epithelial organoids from human colon, adenoma, adenocarcinoma, and Barrett's epithelium. Gastroenterology 141, 1762–1772. 21889923 10.1053/j.gastro.2011.07.050

[28] Marx‐Blumel L , Marx C , Kuhne M & Sonnemann J (2017) Assessment of HDACi‐induced cytotoxicity. Methods Mol Biol 1510, 23–45. 27761811 10.1007/978-1-4939-6527-4_3

[29] Sonnemann J , Marx C , Becker S , Wittig S , Palani CD , Krämer OH & Beck JF (2014) p53‐dependent and p53‐independent anticancer effects of different histone deacetylase inhibitors. Br J Cancer 110, 656–667. 24281001 10.1038/bjc.2013.742 PMC3915118

[30] Brandl A , Wagner T , Uhlig KM , Knauer SK , Stauber RH , Melchior F , Schneider G , Heinzel T & Krämer OH (2012) Dynamically regulated sumoylation of HDAC2 controls p53 deacetylation and restricts apoptosis following genotoxic stress. J Mol Cell Biol 4, 284–293. 22493095 10.1093/jmcb/mjs013

[31] Fujiki Y , Fowler S , Shio H , Hubbard AL & Lazarow PB (1982) Polypeptide and phospholipid composition of the membrane of rat liver peroxisomes: comparison with endoplasmic reticulum and mitochondrial membranes. J Cell Biol 93, 103–110. 7068748 10.1083/jcb.93.1.103 PMC2112093

[32] Poburski D & Thierbach R (2016) Improvement of the BALB/c‐3T3 cell transformation assay: a tool for investigating cancer mechanisms and therapies. Sci Rep 6, 32966. 27611302 10.1038/srep32966 PMC5017208

[33] Westermann M , Steiniger F & Richter W (2005) Belt‐like localisation of caveolin in deep caveolae and its re‐distribution after cholesterol depletion. Histochem Cell Biol 123, 613–620. 15889267 10.1007/s00418-004-0750-5

[34] Olsen JV , de Godoy LMF , Li G , Macek B , Mortensen P , Pesch R , Makarov A , Lange O , Horning S & Mann M (2005) Parts per million mass accuracy on an Orbitrap mass spectrometer via lock mass injection into a C‐trap. Mol Cell Proteomics 4, 2010–2021. 16249172 10.1074/mcp.T500030-MCP200

[35] Keller A , Nesvizhskii AI , Kolker E & Aebersold R (2002) Empirical statistical model to estimate the accuracy of peptide identifications made by MS/MS and database search. Anal Chem 74, 5383–5392. 12403597 10.1021/ac025747h

[36] Frewen B & MacCoss MJ (2007) Using BiblioSpec for creating and searching tandem MS peptide libraries. Curr Protoc Bioinformatics Chapter 13, Unit 13.7. 10.1002/0471250953.bi1307s20 18428681

[37] Reinhold WC , Sunshine M , Liu H , Varma S , Kohn KW , Morris J , Doroshow J & Pommier Y (2012) Cell Miner: a web‐based suite of genomic and pharmacologic tools to explore transcript and drug patterns in the NCI‐60 cell line set. Cancer Res 72, 3499–3511. 22802077 10.1158/0008-5472.CAN-12-1370 PMC3399763

[38] Li T , Fu J , Zeng Z , Cohen D , Li J , Chen Q , Li B & Liu XS (2020) TIMER2.0 for analysis of tumor‐infiltrating immune cells. Nucleic Acids Res 48, W509–W514. 32442275 10.1093/nar/gkaa407 PMC7319575

[39] Li M & Izpisua Belmonte JC (2019) Organoids – preclinical models of human disease. N Engl J Med 380, 569–579. 30726695 10.1056/NEJMra1806175

[40] Trenner A & Sartori AA (2019) Harnessing DNA double‐strand break repair for cancer treatment. Front Oncol 9, 1388. 31921645 10.3389/fonc.2019.01388 PMC6921965

[41] Kopp B , Khoury L & Audebert M (2019) Validation of the gammaH2AX biomarker for genotoxicity assessment: a review. Arch Toxicol 93, 2103–2114. 31289893 10.1007/s00204-019-02511-9

[42] Martini G , Troiani T , Cardone C , Vitiello P , Sforza V , Ciardiello D , Napolitano S , Della Corte CM , Morgillo F , Raucci A *et al*. (2017) Present and future of metastatic colorectal cancer treatment: a review of new candidate targets. World J Gastroenterol 23, 4675–4688. 28765689 10.3748/wjg.v23.i26.4675 PMC5514633

[43] Vaish V , Khare T , Verma M & Khare S (2015) Epigenetic therapy for colorectal cancer. Methods Mol Biol 1238, 771–782. 25421691 10.1007/978-1-4939-1804-1_40

[44] Brooks CL & Gu W (2011) The impact of acetylation and deacetylation on the p53 pathway. Protein Cell 2, 456–462. 21748595 10.1007/s13238-011-1063-9 PMC3690542

[45] Reed SM & Quelle DE (2014) p53 acetylation: regulation and consequences. Cancers (Basel) 7, 30–69. 25545885 10.3390/cancers7010030 PMC4381250

[46] Tang Y , Zhao W , Chen Y , Zhao Y & Gu W (2008) Acetylation is indispensable for p53 activation. Cell 133, 612–626. 18485870 10.1016/j.cell.2008.03.025 PMC2914560

[47] Zheng S , Koh XY , Goh HC , Rahmat SAB , Hwang LA & Lane DP (2017) Inhibiting p53 acetylation reduces cancer chemotoxicity. Cancer Res 77, 4342–4354. 28655792 10.1158/0008-5472.CAN-17-0424

[48] Krummel KA , Lee CJ , Toledo F & Wahl GM (2005) The C‐terminal lysines fine‐tune P53 stress responses in a mouse model but are not required for stability control or transactivation. Proc Natl Acad Sci USA 102, 10188–10193. 16006521 10.1073/pnas.0503068102 PMC1177381

[49] Laptenko O , Shiff I , Freed‐Pastor W , Zupnick A , Mattia M , Freulich E , Shamir I , Kadouri N , Kahan T , Manfredi J *et al*. (2015) The p53 C terminus controls site‐specific DNA binding and promotes structural changes within the central DNA binding domain. Mol Cell 57, 1034–1046. 25794615 10.1016/j.molcel.2015.02.015 PMC6221458

[50] Kon N , Churchill M , Li H , Mukherjee S , Manfredi JJ & Gu W (2021) Robust p53 stabilization is dispensable for its activation and tumor suppressor function. Cancer Res 81, 935–944. 33323382 10.1158/0008-5472.CAN-20-1804 PMC8026563

[51] Friedrich D , Friedel L , Finzel A , Herrmann A , Preibisch S & Loewer A (2019) Stochastic transcription in the p53‐mediated response to DNA damage is modulated by burst frequency. Mol Syst Biol 15, e9068. 31885199 10.15252/msb.20199068 PMC6886302

[52] Tomicic MT , Christmann M & Kaina B (2005) Topotecan‐triggered degradation of topoisomerase I is p53‐dependent and impacts cell survival. Cancer Res 65, 8920–8926. 16204064 10.1158/0008-5472.CAN-05-0266

[53] Schäfer C , Göder A , Beyer M , Kiweler N , Mahendrarajah N , Rauch A , Nikolova T , Stojanovic N , Wieczorek M , Reich TR *et al*. (2017) Class I histone deacetylases regulate p53/NF‐kappaB crosstalk in cancer cells. Cell Signal 29, 218–225. 27838375 10.1016/j.cellsig.2016.11.002

[54] Shukla S , Saxena S , Singh BK & Kakkar P (2017) BH3‐only protein BIM: an emerging target in chemotherapy. Eur J Cell Biol 96, 728–738. 29100606 10.1016/j.ejcb.2017.09.002

[55] Happo L , Cragg MS , Phipson B , Haga JM , Jansen ES , Herold MJ , Dewson G , Michalak EM , Vandenberg CJ , Smyth GK *et al*. (2010) Maximal killing of lymphoma cells by DNA damage‐inducing therapy requires not only the p53 targets Puma and Noxa, but also Bim. Blood 116, 5256–5267. 20829369 10.1182/blood-2010-04-280818 PMC3012543

[56] Zhu H , Swami U , Preet R & Zhang J (2020) Harnessing DNA replication stress for novel cancer therapy. Genes (Basel) 11, 990. 10.3390/genes11090990 PMC7564951 32854236

[57] Perez‐Riverol Y , Csordas A , Bai J , Bernal‐Llinares M , Hewapathirana S , Kundu DJ , Inuganti A , Griss J , Mayer G , Eisenacher M *et al*. (2019) The PRIDE database and related tools and resources in 2019: improving support for quantification data. Nucleic Acids Res 47, D442–D450. 30395289 10.1093/nar/gky1106 PMC6323896

