# Supplementary figures and images for: Mechanistic insights into p53‐regulated cytotoxicity of combined entinostat and irinotecan against colorectal cancer cells
Source: Mol Oncol. 2021 Jul 29;15(12):3404–29. doi: 10.1002/1878-0261.13060 (PMC8637561; doi:10.1002/1878-0261.13060)

# Supplementary figure S1

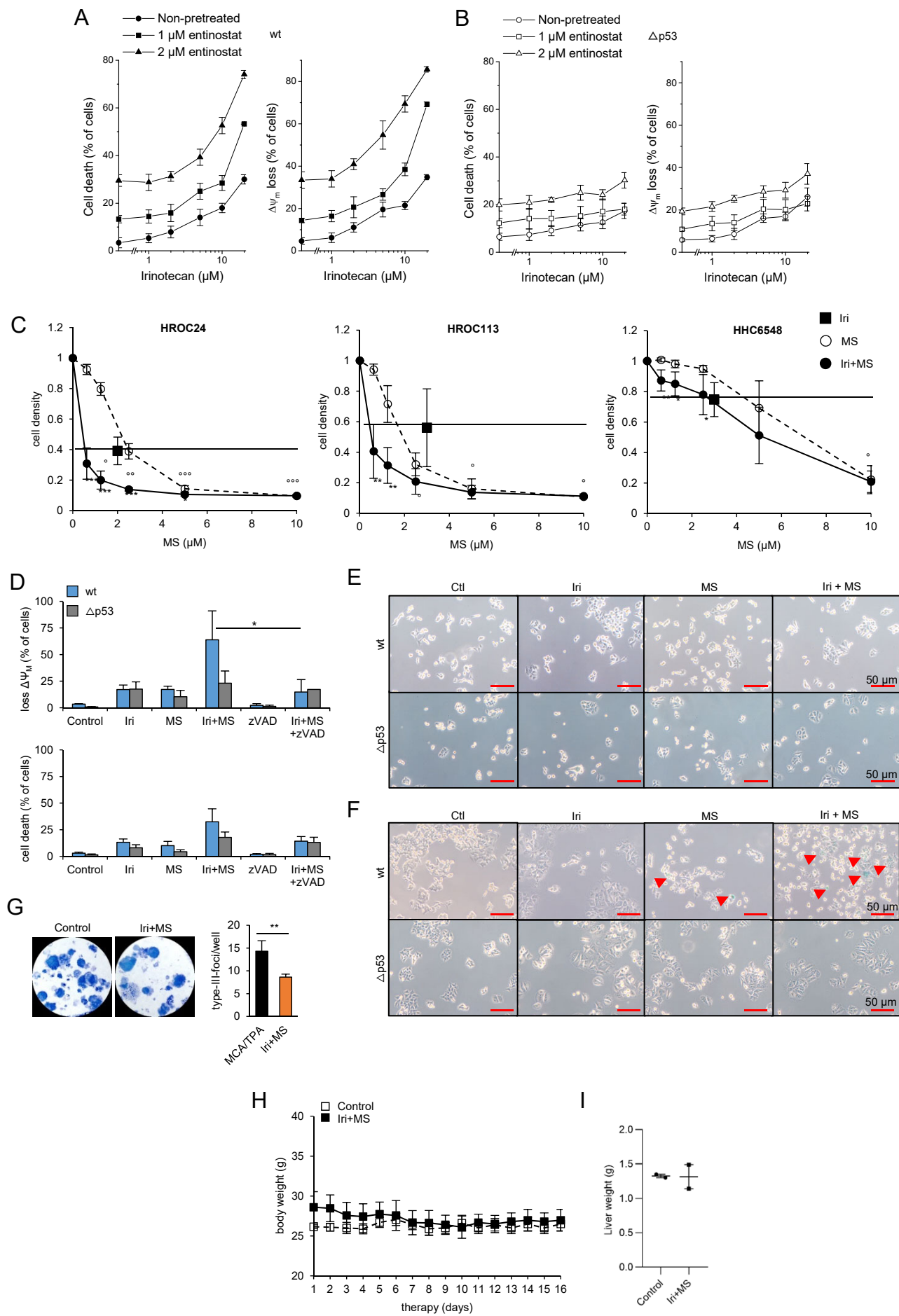

Supplement: Supplementary file 1 — Fig. S1. Irinotecan plus entinostat is effective against CRC cells and tolerated by mice.HCT116wt (A) and HCT116Δp53 (B) cells were exposed to 1‐20 µM irinotecan (Iri) ± 1‐2µM entinostat (MS‐275, abbreviated as MS). Cell death and the loss of ΔΨM were quantified by flow cytometry after 48‐h treatment periods. (C) CRC short‐term cultures were incubated with 0,5‐10 µM MS and irinotecan for 48h. HROC24 cells were treated with 2 µM Iri and HROC113/HHC6548 cells with 3 µM Iri. All graphs in (C) show the average of 3 independent experiments ± SEM; significances for this figure are: * p<0.05; ** p<0.01; *** p<0.001 to MS‐275; ° p<0.05; °° p<0.01; °°° p<0.001 to irinotecan using two‐tailed t‐tests. HCT116wt and HCT116Δp53 cells were exposed to 10 µM Iri ± 2 µM MS. Additionally, cells were 1h pretreated with 20 µM zVAD alone and ± Iri/MS. (D) The loss of ΔΨM and cell death were quantified by flow cytometry after 48h. Cellular senescence was analyzed by β‐galactosidase staining after 24h (E) and 48h (F). Red arrows indicate β‐galactosidase‐positive cells. (G) Representative images of a BALB/c cell transformation assay after 72h of treatment with 10 µM Iri + 2 µM MS. MCA/TPA‐transformed cell foci are Giemsa stained and appear in blue. Quantification of the number of malignancy‐associated type‐III foci/well is shown. All figures/graphs are representative/show the mean value of 3 independent experiments ± SEM; significances for these figure are: * p<0.05; ** p<0.01; *** p<0.001 using two‐tailed t‐tests. (H,I) 6‐ to 8‐week‐old NMRI‐Foxn1nu mice were treated with 20mg/kg Iri and 2,5mg/kg MS for 16 days. (H) Changes in their body weights were monitored daily. (I) The weight of whole livers was documented after 16 days of therapy and are shown as mean values ± SEM. [file MOL2-15-3404-s006.pdf]

Supplementary figure S2

A

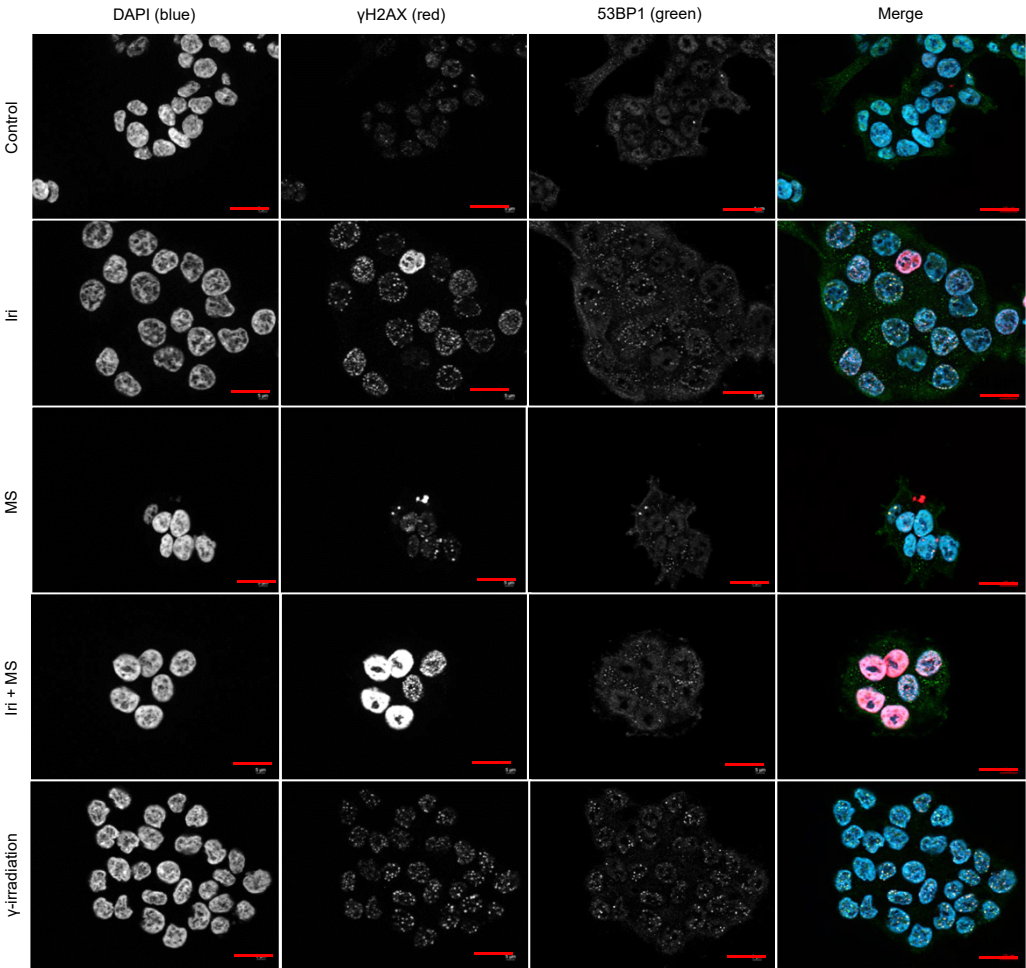

C

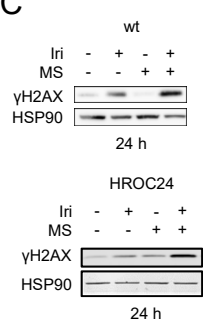

B

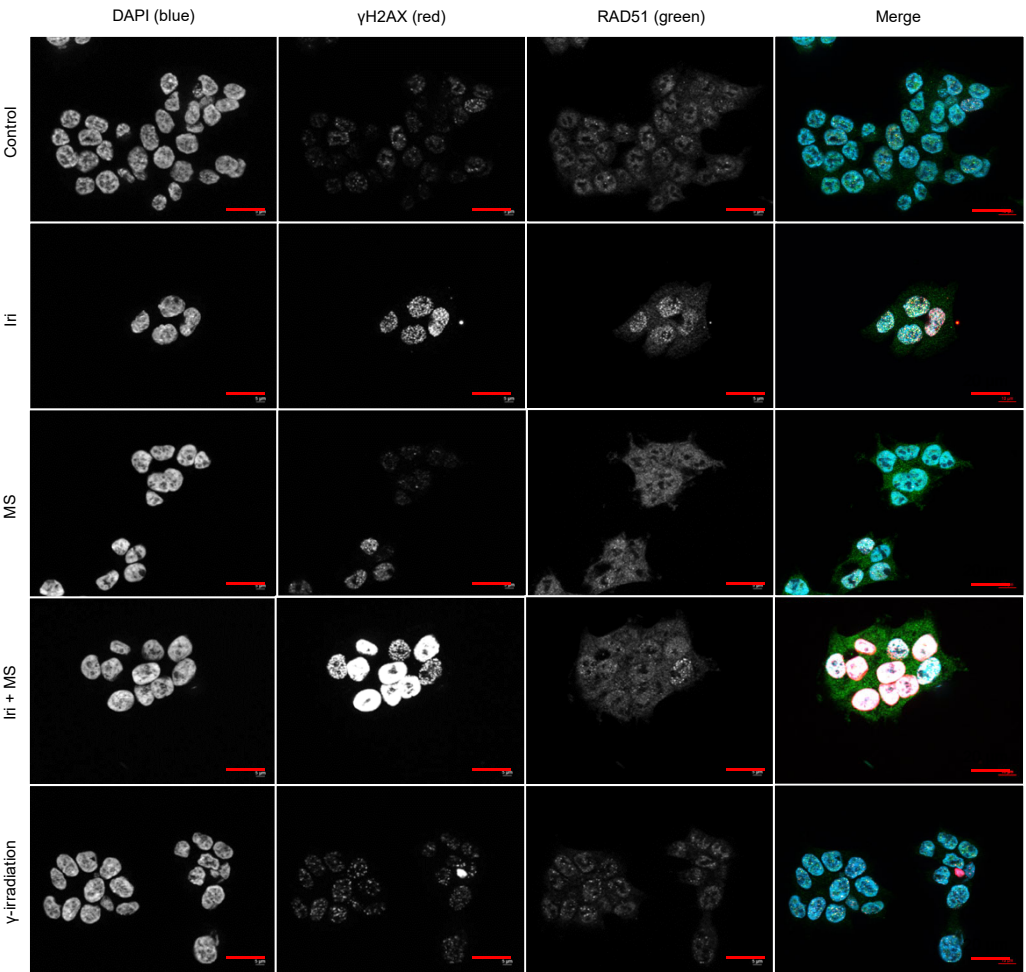

Supplement: Supplementary file 2 — Fig. S2. Irinotecan plus entinostat induces DNA damage in p53 wild‐type CRC cells. HCT116wt cells were exposed to 10 µM irinotecan (Iri) ± 2 µM entinostat/MS‐275 (MS) for 24h or irradiated with 2Gy for 2h before fixation. The expression and localization of 53BP1 (A) or RAD51 (B) together with γH2AX (red) was analyzed by fluorescence microscopy. DAPI was used as nuclear stain. Single channels as well as merged pictures are shown. Pictures are representative for 3 individual experiments. (C) HCT116wt and HROC24 cells were exposed to 5 µM Iri ± 2 µM MS for 24h. Levels of indicated proteins cells were analyzed by immunoblot; HSP90 served as loading control. Immunoblots are representative for 2 independent experiments. [file MOL2-15-3404-s007.pdf]

Supplementary figure S3

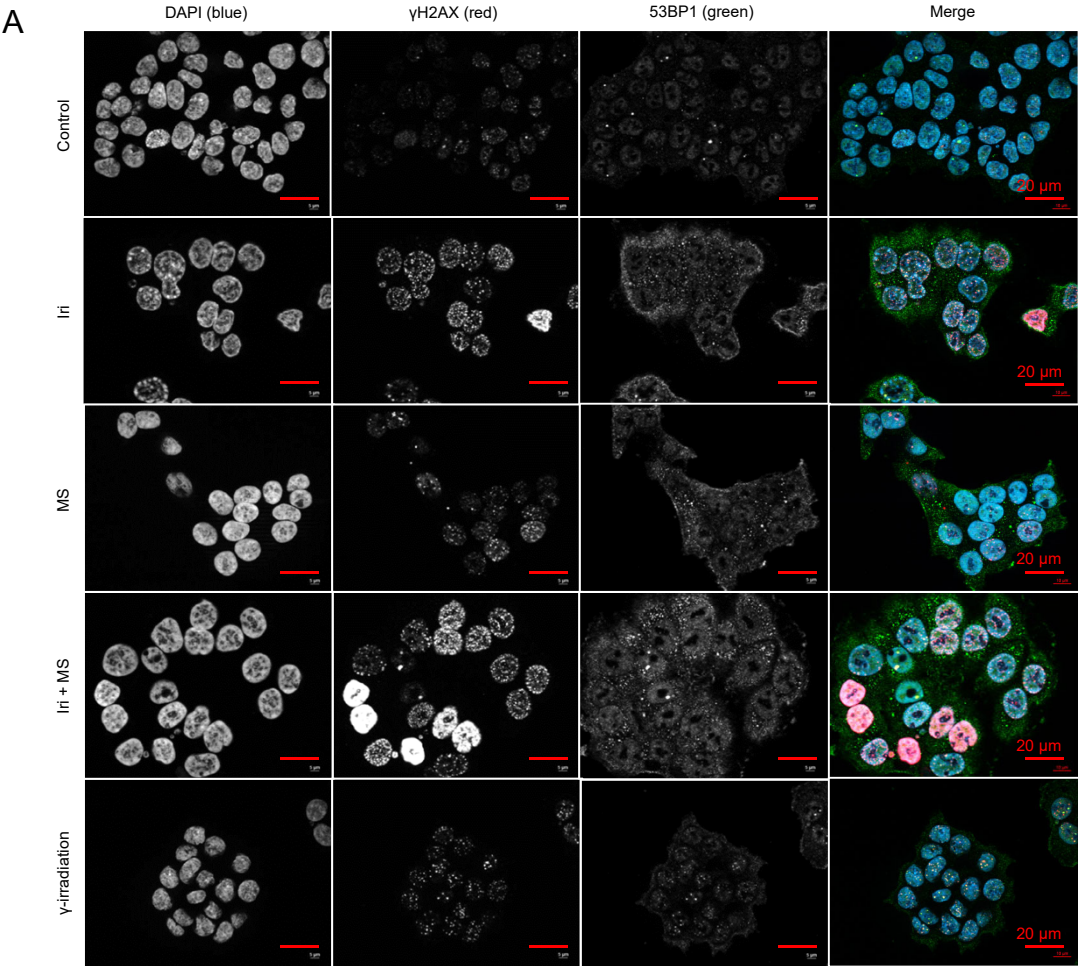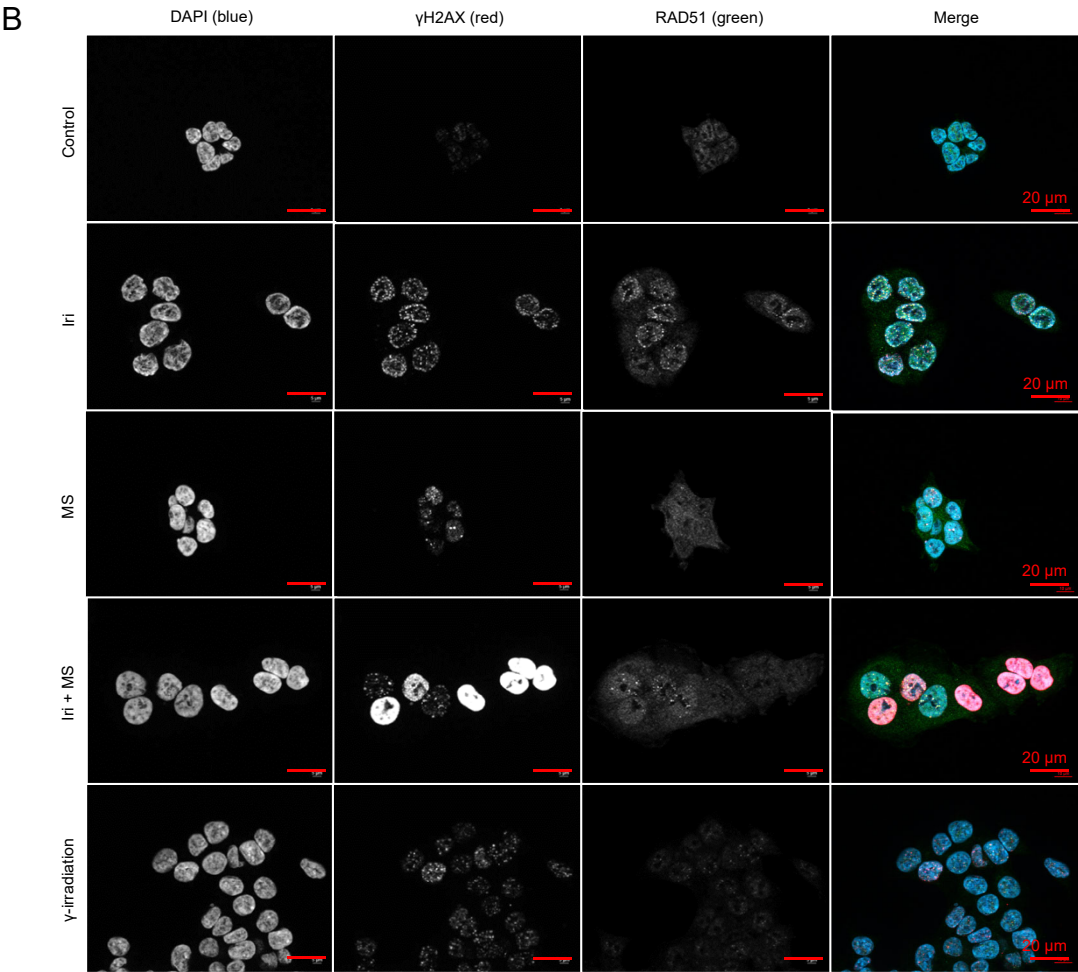

Supplement: Supplementary file 3 — Fig. S3. Irinotecan plus entinostat induces DNA damage in p53 null CRC cells. HCT116Δp53 cells were exposed to 10 µM irinotecan (Iri) ± 2 µM entinostat/MS‐275 (MS) for 24h or irradiated with 2Gy for 2h before fixation. The expression and localization of 53BP1 (A) or RAD51 (B) together with γH2AX (red) was analyzed by fluorescence microscopy. DAPI was used as nuclear stain. Single channels as well as merged pictures are shown. Pictures are representative for 3 individual experiments. [file MOL2-15-3404-s011.pdf]

Supplementary figure S4

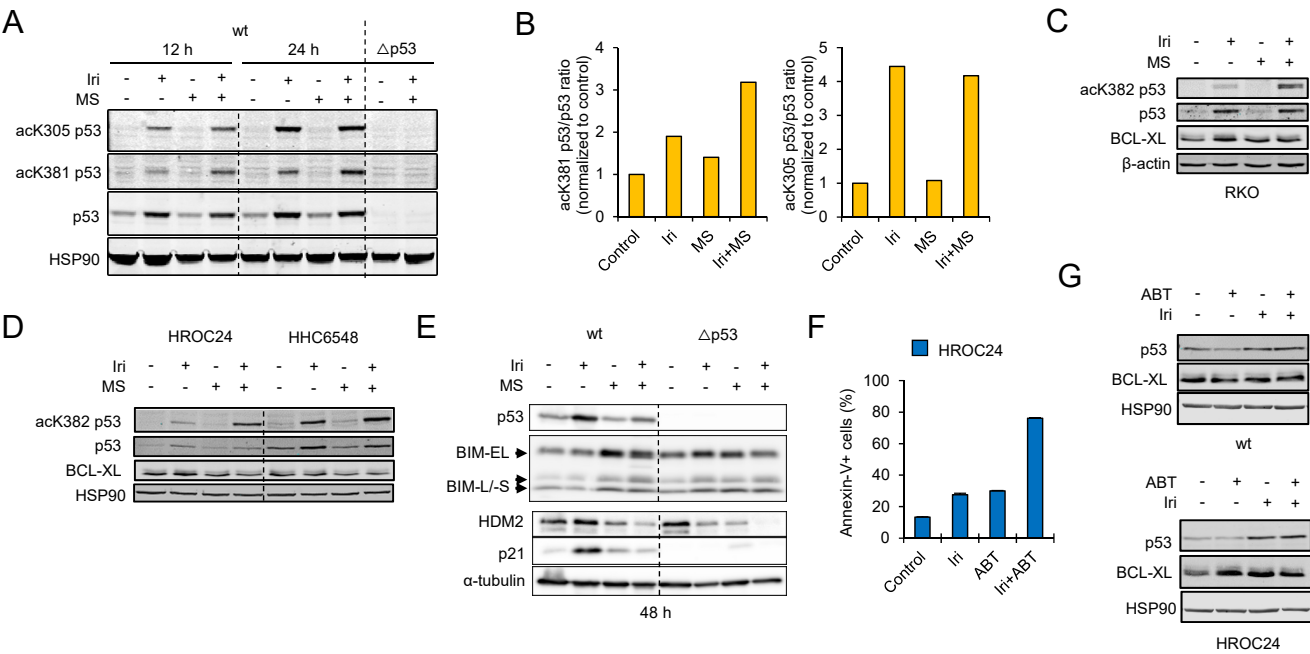

Supplement: Supplementary file 4 — Fig. S4. Irinotecan plus entinostat increases the C‐terminal acetylation of p53 in CRC cells. HCT116wt and HCT116Δp53 cells were exposed to 10 µM irinotecan (Iri) ± 2 µM entinostat/MS‐275 (MS) for 24h. (A) Protein expression levels of indicated proteins and acetylation of p53 were analyzed by immunoblot and quantified using LiCor Odyssey Software; the ratios of acetylated over total p53 were calculated (B). HSP90 served as loading control. RKO (C), HROC24, and HHC6548 (D) cells were exposed to 5 µM Iri ± 2 µM MS. Protein expression levels of indicated proteins and acetylation of p53 were analyzed by immunoblot after 24‐h treatment periods. HSP90 and β‐actin served as loading control. HCT116wt and HCT116Δp53 cells were exposed to 10 µM Iri ± 2 µM MS. (E) Levels of indicated proteins were analyzed by immunoblot after 48h; α‐tubulin served as loading control. HCT116wt and HROC24 cells were exposed to 5 µM Iri ± 500nM navitoclax (ABT‐263, abbreviated as ABT). (F) The percentage of annexin‐V‐positive (i.e., apoptotic) HROC24 cells was quantified by flow cytometry after 48h. (G) Levels of indicated proteins were analyzed by immunoblot after 24h; HSP90 served as loading control. (A‐B) show 1 representative experiment each (C‐G) are representative for/show the mean value of 2 individual experiments. [file MOL2-15-3404-s008.pdf]

Supplementary figure S5

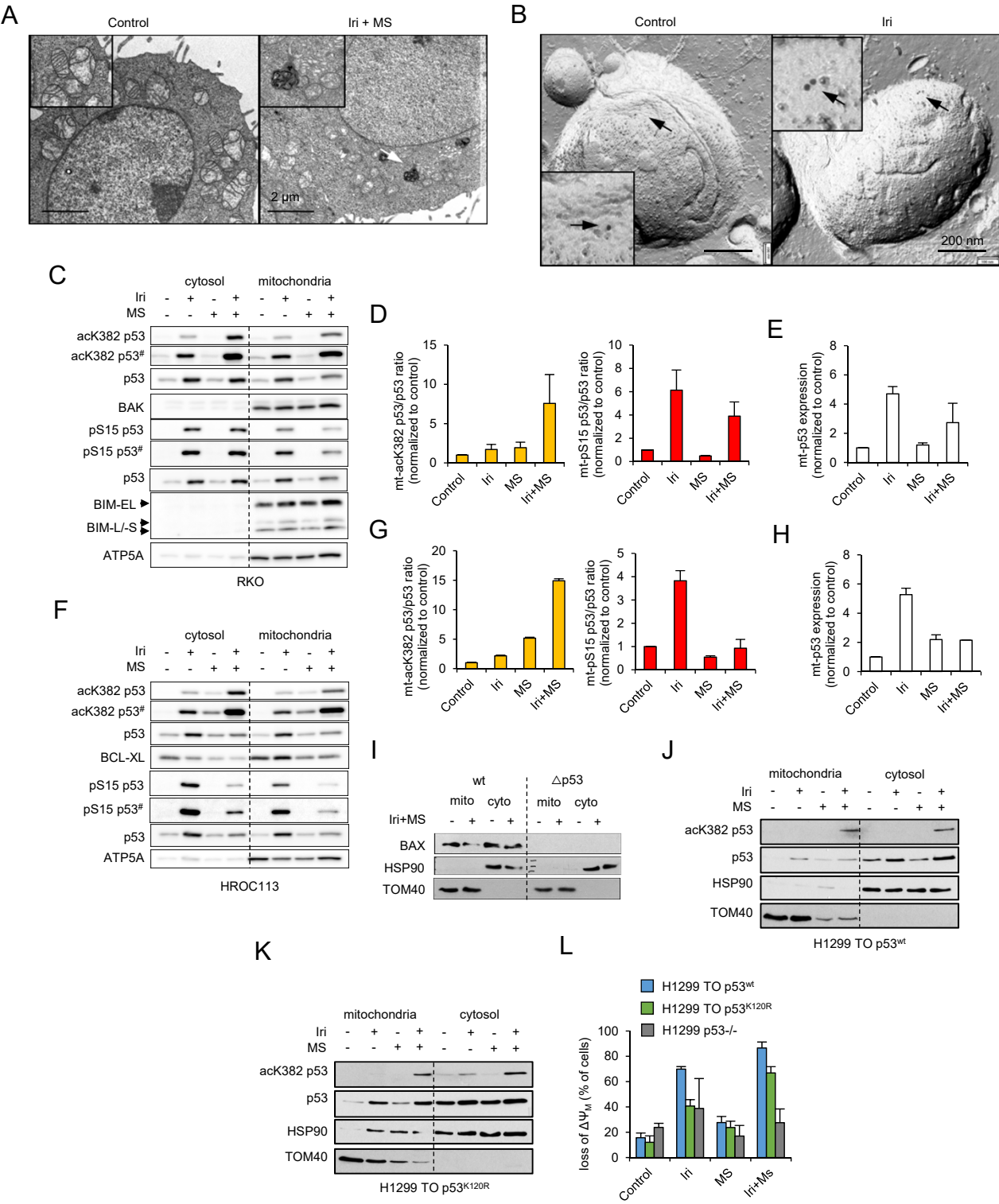

Supplement: Supplementary file 5 — Fig. S5. C‐terminally acetylated p53 locates at mitochondria after treatment with irinotecan plus entinostat. HCT116wt and HCT116Δp53 cells were exposed to 10 µM irinotecan (Iri) ± 2 µM entinostat/MS‐275 (MS) for 24h. (A) Mitochondrial morphologies in HCT116Δp53 cells were analyzed by TEM. Representative mitochondria are shown enlarged in the top left corner of each panel. Black arrows indicate damaged mitochondria. (B) Localization of immune‐gold‐labeled p53 was analyzed by TEM on freeze‐fractures from HCT116wt cells. Gold particles are indicated by black arrows and shown enlarged in the bottom left corner of each panel. RKO (C‐E) and HROC113 (F‐H) cells were exposed to 10 µM Iri ± 2 µM MS for 24h. Cell lysates were fractionated into mitochondria and cytosol. The expression of indicated proteins, localization, and modification of p53 in RKO (C) and HROC113 cells (F) was analyzed by immunoblot. ATP5A served as mitochondrial protein loading. Quantification of corresponding p53 PTMs was done by ImageJ; the ratios of mitochondrial (mt)‐pS15‐ and mt‐acK382‐ over total p53 in RKO (D) and HRCO113 cells (G), were calculated. Quantification of mitochondrial total p53 level is shown in (E) for RKO and in (H) for HROC113 cells. HCT116wt and HCT116Δp53 cells were exposed to 10 µM Iri ± 2 µM MS for 24h. (I) Cell lysates were fractionated into mitochondria and cytosol. The expression of indicated proteins was analyzed by immunoblot. HSP90 and TOM40 served as protein loading control within individual compartments. Tetracyclin‐induced H1299 TO p53wt or H1299 TO p53K120R cells were exposed to 10 µM Iri ± 2 µM MS. (J‐K) Cell lysates were fractionated into mitochondria and cytosol after 24h. The expression, localization, and acetylation of p53 was analyzed by Immunoblot. HSP90 and TOM40 served as protein loading control within individual compartments. (L) The loss of ΔΨM was quantified by flow cytometry after 48h. (A‐L) are representative for/show the mean value of 2 individual exper [file MOL2-15-3404-s002.pdf]

# Supplementary figure S6

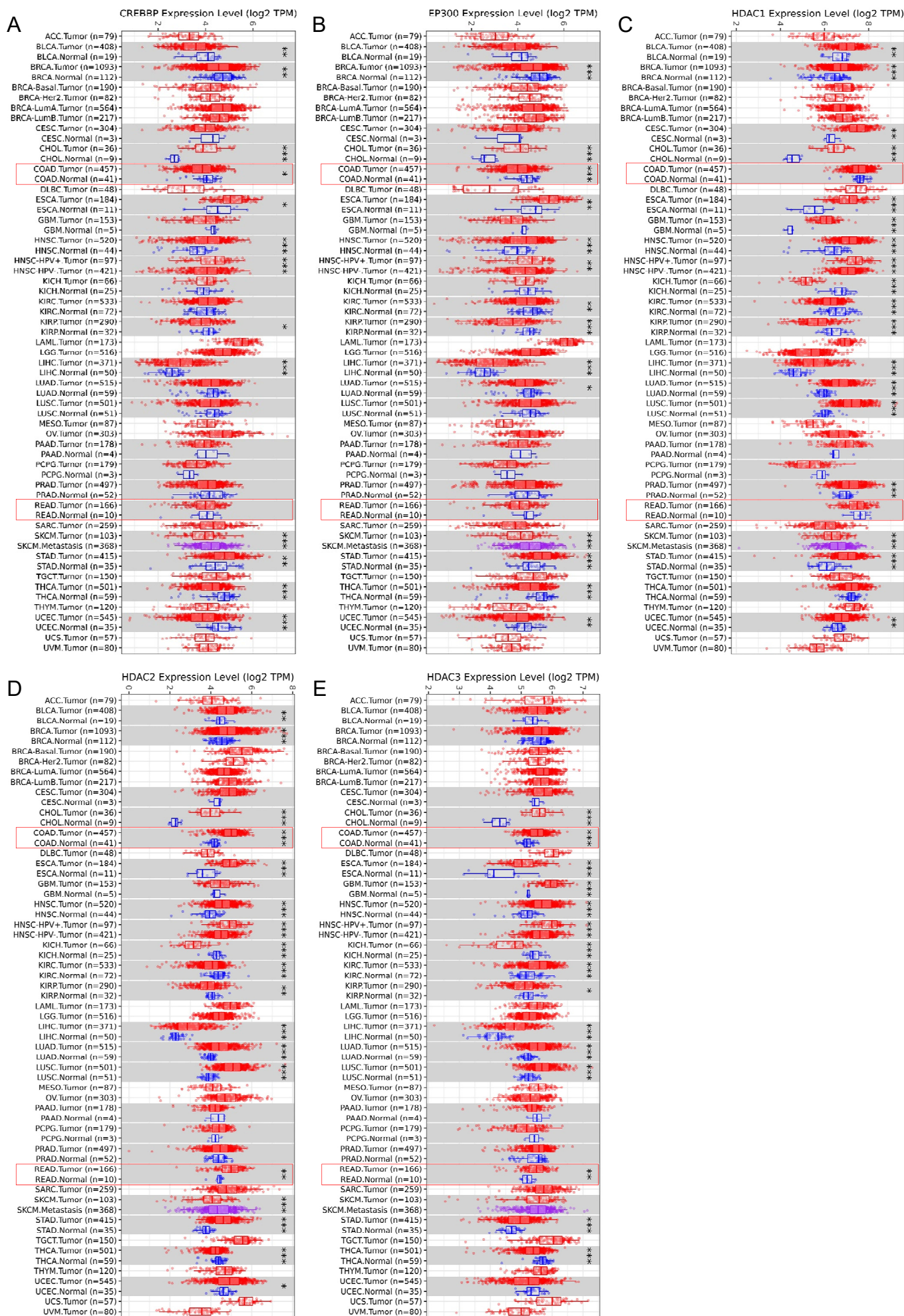

Supplement: Supplementary file 6 — Fig. S6. Differential gene expression of HATs and HDACs in human cancer samples. Gene expression of The Cancer Genome Atlas (TCGA) datasets was analyzed for indicated genes using Gene DE module in the Timer web platform. Differences in gene expression level are depicted as log2‐fold change. Colon adenocarcinomas (COAD) and rectum adenocarcinomas (READ) are highlighted. Significances for this figure are: * p<0.05; ** p<0.01; *** p<0.001. [file MOL2-15-3404-s004.pdf]

Supplementary figure S7

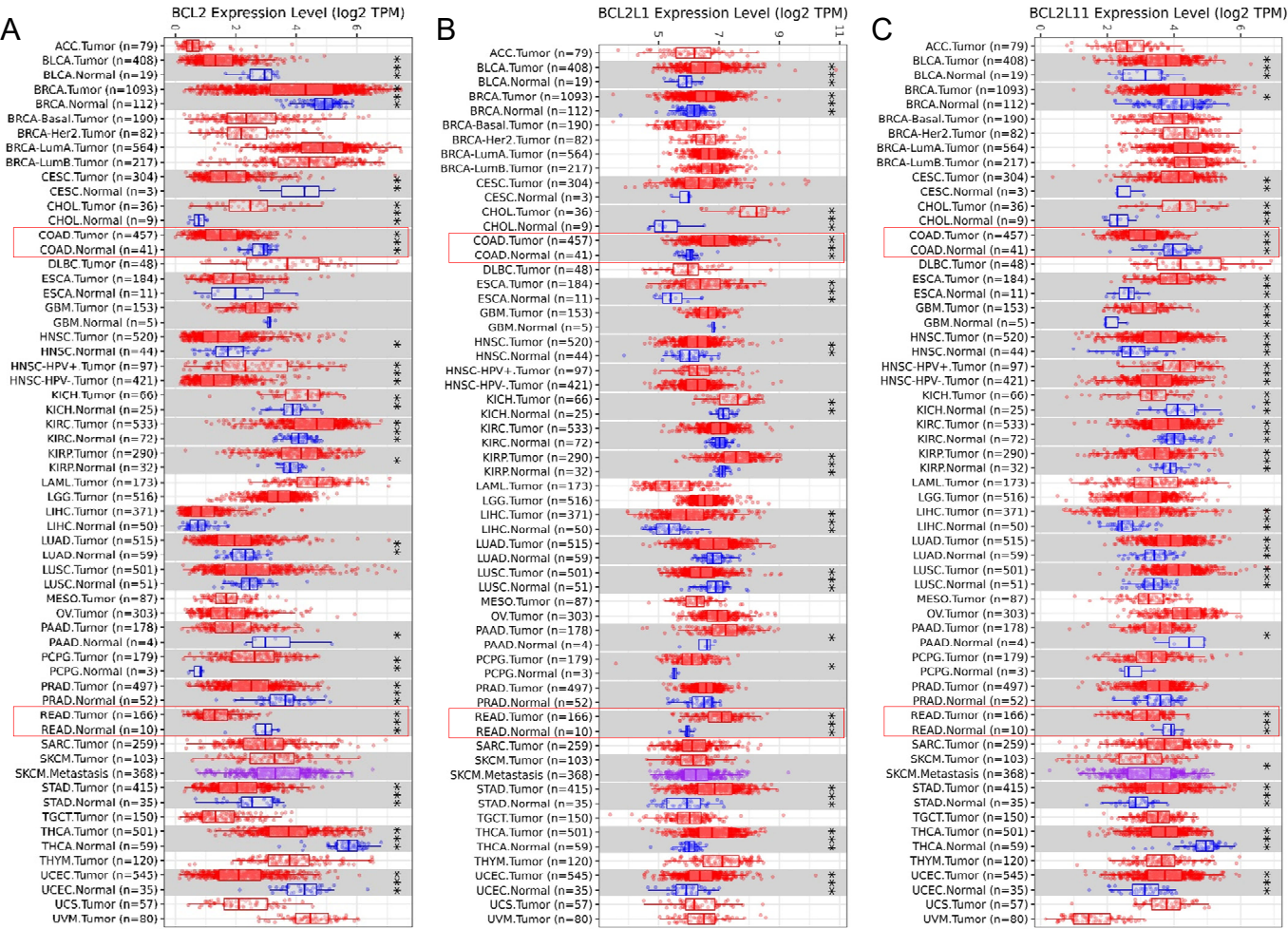

Supplement: Supplementary file 7 — Fig. S7. Differential gene expression of BCL2 proteins in human cancer samples. Gene expression of The Cancer Genome Atlas (TCGA) datasets was analyzed for indicated genes using Gene DE module in the Timer web platform. Differences in gene expression level are depicted as log2‐fold change. Colon adenocarcinomas (COAD) and rectum adenocarcinomas (READ) are highlighted. Significances for this figure are: * p<0.05; ** p<0.01; *** p<0.001. [file MOL2-15-3404-s003.pdf]

Supplementary figure S8

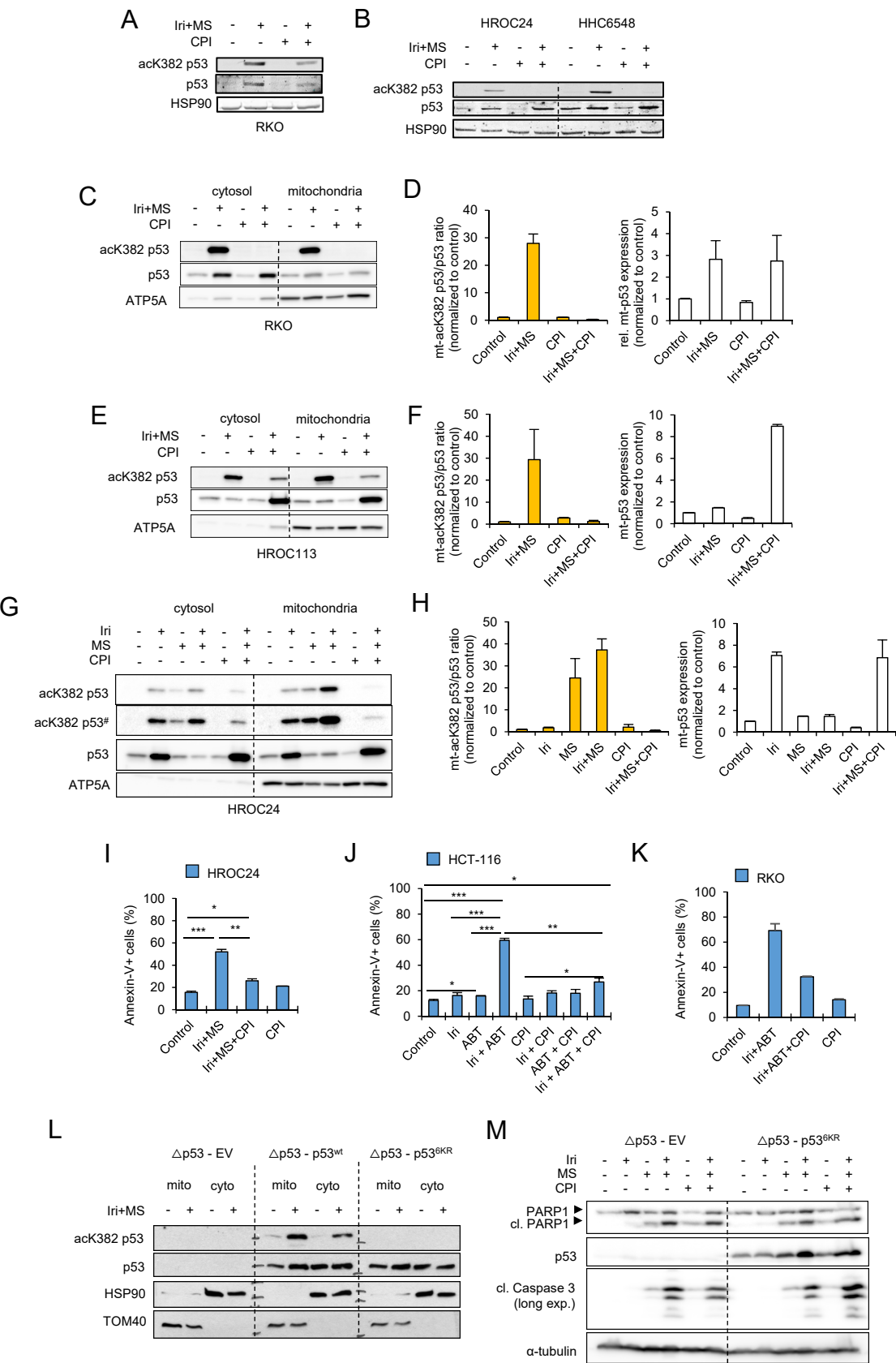

Supplement: Supplementary file 8 — Fig. S8. C‐terminally acetylated p53 is necessary to induce apoptosis by irinotecan plus entinostat. RKO (A), HROC24, and HHC6548 (B) cells were exposed to 5 µM irinotecan (Iri) + 2 µM MS‐275 (MS) ± 200nM CPI‐1612 (CPI). Levels of indicated proteins and acetylation of p53 were analyzed by immunoblot after 24h. HSP90 served as loading control. RKO (C‐D) and HROC113 (E‐F) cells were exposed to 10 µM Iri + 2 µM MS ± 200nM CPI. Cell lysates were fractionated into mitochondria and cytosol after 24h. The expression of indicated proteins, localization, and acetylation of p53 in RKO (C) and HROC113 cells (E) was analyzed by immunoblot. ATP5A served as mitochondrial protein loading. Quantification of p53 acetylation was done by ImageJ; the ratios of mitochondrial mt‐acK382‐ over total p53 in RKO (D) and HRCO113 cells (F) were calculated and the expression of total p53 is shown correspondingly. (G‐H) HROC24 cells were exposed to 10 µM Iri ± 2 µM MS ± 200nM CPI. Cell lysates were fractionated into mitochondria and cytosol after 24h. The expression of indicated proteins, localization, and acetylation of p53 was analyzed by immunoblot (G). ATP5A served as mitochondrial protein loading. Quantification of p53 acetylation was done by ImageJ; the ratios of mitochondrial mt‐acK382‐ over total p53 (H) were calculated and the expression of mitochondrial total p53 is shown correspondingly. (I) HROC24 cells were exposed to 5 µM Iri + 2 µM MS ± 200nM CPI for 48h. The percentage of apoptotic Annexin‐V‐positive was quantified by flow cytometry afterward. (J) HCT116wt cells were exposed to 5 µM Iri ± 500nM ABT‐263 (ABT) ± 200nM CPI for 48h. The percentage of apoptotic Annexin‐V‐positive was quantified by flow cytometry afterward. (K) RKO cells were exposed to 5 µM Iri + 500nM ABT ± 200nM CPI for 72h. The percentage of apoptotic Annexin‐V‐positive was quantified by flow cytometry afterward. (L) HCT116Δ p53 were transient transfected with empty vector (Δp53 ‐ EV) for control, wild‐type p53 (Δp [file MOL2-15-3404-s005.pdf]

Supplementary figure S9

A

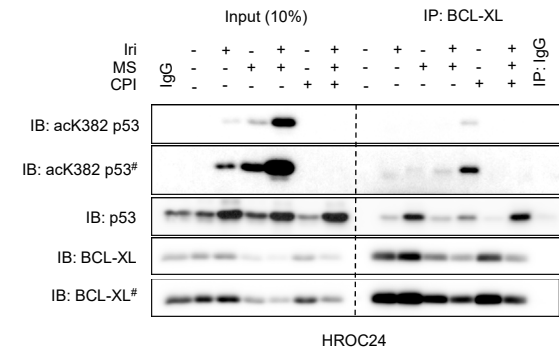

B

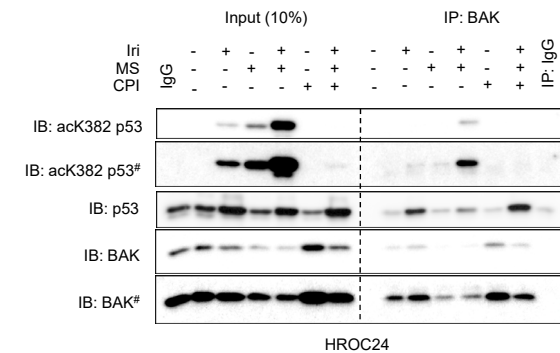

C

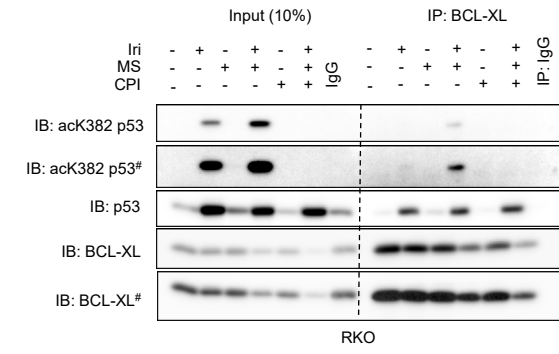

D

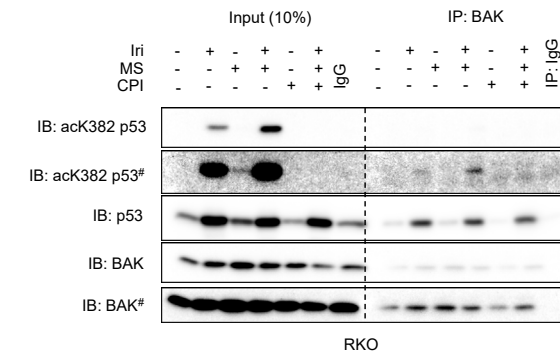

Supplement: Supplementary file 9 — Fig. S9. C‐terminally acetylated p53 interacts with BAK to promote apoptosis. HROC24 cells were exposed to 5 µM irinotecan (Iri) ± 2 µM entinostat/MS‐275 (MS) ± 200nM CPI‐1612 (CPI). BCL‐XL (A) and BAK (B) were immunoprecipitated from cell lysates and expression levels of indicated proteins and acetylation of p53 were analyzed by immunoblot afterward. RKO cells were exposed to 10 µM Iri ± 2 µM MS ± 200nM CPI. BCL‐XL (C) and BAK (D) were immunoprecipitated from cell lysates and expression levels of indicated proteins and acetylation of p53 were analyzed by immunoblot afterward. All immunoblots and graphs are representative of 2 independent experiments. # indicate long exposures of corresponding proteins for immunoblots. [file MOL2-15-3404-s012.pdf]
