# Supplementary material for: Mechanistic insights into p53‐regulated cytotoxicity of combined entinostat and irinotecan against colorectal cancer cells
Source: Mol Oncol. 2021 Jul 29;15(12):3404–29. doi: 10.1002/1878-0261.13060 (PMC8637561; doi:10.1002/1878-0261.13060)
Supplement: Supplementary file 10 — Table S1. Synergistic interaction of irinotecan plus entinostat combinations in CRC cells. HCT116wt and HCT116Δp53 cells were exposed to 1‐20 µM irinotecan (Iri) ± 1‐2 µM entinostat (MS‐275, abbreviated as MS). Cell death and the loss of ΔΨM were quantified by flow cytometry after 48h treatment periods. CI‐values were calculated with CalcuSyn from these dose‐response curves (see Figures S1A‐B). [file MOL2-15-3404-s010.pdf]

[MS-275] (μM)	[Irinotecan] (μM)	wt CI	△p53 CI
1	1	1.120	0.917
1	2	1.170	0.995
1	5	0.907	1.066
1	10	1.008	1.052
1	20	0.449	1.254
2	1	1.076	0.947
2	2	1.023	0.888
2	5	0.857	0.744
2	10	0.614	0.845
2	20	0.316	0.584
