# Supplementary material for: Mechanistic insights into p53‐regulated cytotoxicity of combined entinostat and irinotecan against colorectal cancer cells
Source: Mol Oncol. 2021 Jul 29;15(12):3404–29. doi: 10.1002/1878-0261.13060 (PMC8637561; doi:10.1002/1878-0261.13060)
Supplement: Supplementary file 11 — Table S2. Differential gene expression of HATs and HDACs in human cancer cell lines. Gene expression data of indicated genes in the NCI‐60 cell line panel were exported using the CellMiner web tool. Changes of transcript intensities are depicted as z‐scores. CRC cell lines (CO) are highlighted. [file MOL2-15-3404-s001.pdf]

Average transcript intensity z scores

Cell lines	CREBBP	EP300	HDAC1	HDAC2	HDAC3
BR:MCF7	1.538	-0.954	0.019	-0.723	1.102
BR:MDA-MB-231	-0.425	-0.739	-1.395	-0.418	1.155
BR:HS 578T	-1.13	0.28	-0.786	-0.861	-0.913
BR:BT-549	-0.16	-0.21	-0.657	0.545	-0.514
BR:T-47D	0.855	-0.117	-0.291	-1.738	0.754
CNS:SF-268	0.471	-0.312	-0.106	1.125	1.237
CNS:SF-295	1.154	0.219	-0.228	0.263	1.322
CNS:SF-539	1.213	0.011	0.458	-0.326	0.589
CNS:SNB-19	-0.866	-0.251	0.526	0.805	0.83
CNS:SNB-75	0.627	-0.097	-0.114	-0.711	-0.098
CNS:U251	-0.474	-0.363	1.121	-0.257	0.715
CO:COLO 205	-0.591	-0.028	1.57	-0.195	1.046
CO:HCC-2998	0.399	-0.071	1.166	1.519	0.255
CO:HCT-116	0.348	-0.282	1.264	0.704	1.3
CO:HCT-15	0.35	-1.052	1.106	1.218	0.183
CO:HT29	0.105	-0.758	1.337	-0.322	0.773
CO:KM12	0.659	0.931	1.787	0.075	1.242
CO:SW-620	-0.593	0.326	0.844	1.926	0.3
LE:CCRF-CEM	-0.252	-0.209	0.871	0.444	0.305
LE:HL-60(TB)	-1.871	0.179	0.575	2.026	1.444
LE:K-562	-1.617	-1.103	0.493	-0.126	1.281
LE:MOLT-4	-0.63	-0.453	1.097	0.427	0.307
LE:RPMI-8226	0.762	0.449	-0.726	-0.979	-2.006
LE:SR	-0.002	0.768	0.508	0.822	0.897
ME:LOX IMVI	1.112	0.222	0.249	1.634	0.338
ME:MALME-3M	1.553	1.086	-0.679	-0.29	-1.713
ME:M14	-0.075	1.315	1.178	-1.102	-0.841
ME:SK-MEL-2	0.053	3.844	-0.265	0.862	0.099
ME:SK-MEL-28	-0.641	1.082	0.375	-0.249	-0.672
ME:SK-MEL-5	-0.706	0.955	-0.922	0.376	-0.004
ME:UACC-257	0.122	0.421	0.069	-0.758	-0.375
ME:UACC-62	-0.462	0.622	0.09	-0.319	-0.397
ME:MDA-MB-435	0.42	1.313	-0.297	-0.154	0.115
ME:MDA-N	-0.108	0.902	-0.428	-0.686	-0.473
LC:A549/ATCC	-0.492	-0.706	-1.07	-1.635	-1.374
LC:EKVX	-0.176	-0.059	-1.534	-0.65	-1.102
LC:HOP-62	-0.79	-0.304	0.271	0.136	0.309
LC:HOP-92	-0.956	-0.849	-0.238	-1.313	-1.075
LC:NCI-H226	-1.211	-1.16	-2.201	-1.056	-0.078
LC:NCI-H23	0.527	0.615	0.38	-0.061	1.203
LC:NCI-H322M	0.787	1.148	-0.309	-0.221	-1.039
LC:NCI-H460	-0.486	-0.983	-1.799	-1.127	-0.505
LC:NCI-H522	-0.129	0.06	0.293	0.755	0.664
OV:IGROV1	0.234	0.735	0.298	0.015	-0.19
OV:OVCAR-3	0.485	-0.389	-0.254	1.299	0.332
OV:OVCAR-4	-0.83	-1.021	0.368	-0.508	1.205
OV:OVCAR-5	1.412	1.831	0.834	-0.322	0.586
OV:OVCAR-8	-1.667	-0.215	-0.224	1.224	0.398
OV:SK-OV-3	2.239	0.99	0.225	0.761	0.299
OV:NCI/ADR-RES	-2.032	0.025	-0.599	0.594	-0.299
PR:PC-3	-0.399	-0.574	1.309	0.761	-1.001
PR:DU-145	-0.56	-0.541	-1.542	-0.755	-0.462
RE:786-0	1.036	-0.027	0.607	0.963	0.121
RE:A498	1.061	-1.569	-1.652	-1.048	-0.867
RE:ACHN	0.798	-0.952	-0.75	-0.534	-1.735
RE:CAKI-1	0.124	-1.145	-0.363	-0.803	-1.087
RE:RXF 393	-0.663	0.163	0.346	-1.344	-0.749
RE:SN12C	-0.163	-1.321	-0.106	0.134	-0.247
RE:TK-10	1.169	-0.8	-0.922	0.039	-0.74
RE:UO-31	0.722	-0.855	-0.8	-0.192	-1.581
