# Supplementary material for: Mechanistic insights into p53‐regulated cytotoxicity of combined entinostat and irinotecan against colorectal cancer cells
Source: Mol Oncol. 2021 Jul 29;15(12):3404–29. doi: 10.1002/1878-0261.13060 (PMC8637561; doi:10.1002/1878-0261.13060)
Supplement: Supplementary file 12 — Table S3. Mutations of HAT and HDAC genes in human cancer cell lines. Mutations of indicated genes in the NCI‐60 cell line panel were exported using the CellMiner web tool. The number of mutations as absolute numbers. CRC cell lines (CO) are highlighted. [file MOL2-15-3404-s009.pdf]

Summation of amino acid changing variant(s) by gene

Cell lines	CREBBP	EP300	HDAC1	HDAC2	HDAC3	Sum
BR:MCF7	0	45	0	0	0	45
BR:MDA-MB-231	0	0	0	0	0	0
BR:HS 578T	0	0	0	0	0	0
BR:BT-549	0	0	0	0	0	0
BR:T-47D	0	0	0	0	0	0
CNS:SF-268	0	0	0	0	0	0
CNS:SF-295	0	0	0	0	0	0
CNS:SF-539	0	0	0	0	0	0
CNS:SNB-19	0	0	0	0	0	0
CNS:SNB-75	0	24	0	0	0	91
CNS:U251	0	41	0	0	0	41
CO:COLO 205	0	0	0	0	0	0
CO:HCC-2998	0	83	0	76	0	229
CO:HCT-116	0	49	0	0	0	49
CO:HCT-15	79	100	0	0	0	179
CO:HT29	57	0	0	0	0	57
CO:KM12	58	55	0	0	0	167
CO:SW-620	0	26	0	0	0	54
LE:CCRF-CEM	0	0	0	0	0	0
LE:HL-60(TB)	0	0	0	0	0	0
LE:K-562	0	0	0	0	0	0
LE:MOLT-4	0	0	0	0	0	0
LE:RPMI-8226	0	0	0	0	0	0
LE:SR	0	0	0	0	0	49
ME:LOX IMVI	0	0	0	0	0	0
ME:MALME-3M	0	0	0	0	0	0
ME:M14	0	0	0	0	0	0
ME:SK-MEL-2	0	0	0	0	0	0
ME:SK-MEL-28	0	0	0	0	0	0
ME:SK-MEL-5	0	0	0	0	0	0
ME:UACC-257	0	0	0	0	0	0
ME:UACC-62	0	0	0	0	0	0
ME:MDA-MB-435	0	0	0	0	0	0
ME:MDA-N	0	0	0	0	0	0
LC:A549/ATCC	0	0	0	0	0	0
LC:EKVX	0	0	0	0	0	0
LC:HOP-62	0	0	0	0	0	0
LC:HOP-92	0	100	0	0	0	100
LC:NCI-H226	0	0	0	0	0	0
LC:NCI-H23	0	0	0	0	0	0
LC:NCI-H322M	90	0	100	100	0	290
LC:NCI-H460	0	0	0	0	0	0
LC:NCI-H522	0	0	0	0	0	59
OV:IGROV1	54	0	0	0	0	54
OV:OVCAR-3	0	0	0	0	0	0
OV:OVCAR-4	0	0	0	0	0	0
OV:OVCAR-5	63	21	0	0	0	84
OV:OVCAR-8	100	47	0	0	0	147
OV:SK-OV-3	0	50	0	0	0	50
OV:NCI/ADR-RES	100	62	0	0	0	162
PR:PC-3	0	0	0	0	0	0
PR:DU-145	0	0	0	0	0	0
RE:786-0	0	0	0	0	0	0
RE:A498	0	0	0	0	0	0
RE:ACHN	0	0	0	0	0	0
RE:CAKI-1	31	0	0	0	0	31
RE:RXF 393	0	21	0	0	0	21
RE:SN12C	0	100	0	0	0	100
RE:TK-10	0	0	0	0	0	0
RE:UO-31	0	0	0	0	0	0
